# Effects of Exergaming on executive function and motor ability in children: A systematic review and meta-analysis

**DOI:** 10.1371/journal.pone.0309462

**Published:** 2024-09-06

**Authors:** Ruijie Kou, Zhenjie Zhang, Feilong Zhu, Yanli Tang, Zixuan Li

**Affiliations:** 1 Capital University of Physical Education and Sports, Beijing, China; 2 College of Physical Education and Sports, Beijing Normal University, Beijing, China; 3 School of Psychological and Cognitive Sciences, Peking University, Beijing, China; Padjadjaran University: Universitas Padjadjaran, INDONESIA

## Abstract

This study aimed to evaluate the effectiveness of Exergaming in improving executive function and motor ability across different groups of children and adolescents. We searched several databases, including PsycINFO, Web of Science, Embase, PubMed, SPORT Discus, Scopus, and the Cochrane Central Register of Controlled Trials, for randomized controlled trial (RCT) studies published from inception until November 25, 2023, to identify studies investigating the effect of Exergaming on motor and executive function in children. The protocol was registered with PROSPERO (CRD42023482281). A total of 37 randomized controlled trials were included in this study. Our results indicate that Exergaming can influence children’s cognitive flexibility [SMD = 0.34, 95%CI(0.13,0.55), I^2^ = 0.0%, *P* = 0.738], inhibition control [SMD = 0.51, 95%CI (0.30,0.72), I^2^ = 0.0%, *P* = 0.473], global cognitive [SMD = 0.87, 95%CI (0.50,1.23), I^2^ = 0.0%, *P* = 0.974], working memory [SMD = 0.18, 95%CI(-0.16, 0.52), I^2^ = 46.5%, *P* = 0.096], gross motor skills [SMD = 0.82, 95%CI (0.30, 1.35), I^2^ = 79.1%, *P*<0.001], fine motor skills [SMD = 0.71, 95%CI (0.22,1.21), I^2^ = 78.7%, *P*<0.001], balance [SMD = 0.61, 95%CI (0.34, 0.88), I^2^ = 59.5%, *P* = 0.001], and cardiorespiratory [SMD = 0.48, 95%CI (0.16, 0.79), I^2^ = 58.4%, *P* = 0.019]. While these findings suggest that Exergaming can promote children’s cognitive flexibility, inhibitory control, global cognition and motor abilities, the effect on working memory was not statistically significant. Further high-quality randomized controlled trials are warranted to explore the potential benefits of Exergaming for different groups of children, including those with specific needs.

## Introduction

In 2018, the World Health Organization initiated More Active People for a Healthier World, a new global initiative on physical activity (PA), to enhance physical activity and reduce disease rates among adolescents and adults worldwide. Physical activity has several well-documented health advantages [1], including improved quality of life (QoL), prevention and treatment of disease, and favorable effects on physical, psychological, cognitive, and social conditions [2–5]. For children, physical activity plays an irreplaceable role in their growth and improving their overall fitness level. Current evidence suggests that these health benefits continue from childhood into adulthood [6].

Despite the recognized benefits of physical activity, research published in The Lancet by Guthold et al. in 2020 found that over four out of five teenagers enrolled in school did not engage in enough physical activity [7]. This trend of physical inactivity in adolescents is linked to a rise in closely related physical and psychological problems such as obesity and depression [8, 9]. The proliferation of digital technology has further compounded this issue. In this respect, the widespread usage of the internet, particularly among young teens and college students, has led to a surge in the popularity of video games as a form of entertainment worldwide [10]. Studies have shown a concerning increase in screen time among children. For instance, 30% to 60% of school-age children in Europe spend more than 2h a day playing games on their cell phones, tablets, and other devices [11], there are also reports indicating that 97% of teenagers in the United States play some form of video game [12]. With an estimated 3.2 billion video game players worldwide in 2022, and a projected increase to over 3.6 billion by 2025 [13], this trend has intensified the issue of increased sedentary time and screen time among children, leading to a lack of exercise, and evolving into a global public health challenge [14].

While games can play a crucial role in fostering child development [15], it is also important to acknowledge the potential negative impacts of video games. Video game addiction has been linked to a several negative consequence, including impulsivity, sadness, exhaustion, loneliness, and low self-esteem [16]. In the current digital age, the trend of adolescents spending significant time on screens and video games is unlikely to reverse. Therefore, emphasis should be placed on promoting healthier screen time habits. This could involve encouraging a shift from sedentary video games with minimal physical activity towards active video games that require more movement. By promoting these active video games, we can leverage technology to support the healthy development of adolescents [17].

Exergaming, also known as active video games, has emerged as a promising technology-based exercise program [18]. It refers to video games that require players to use their entire bodies to participate in the gameplay through physical activity [19]. Examples include Wii Fit, Microsoft Xbox Kinect, Wii Sports software, and Dance Dance Revolution [20]. Exergaming is reportedly an effective strategy to encourage more exercise [21, 22]. Engaging in these physically demanding video games can potentially enhance physical activity levels and reduce sedentary habits [23–26]. Studies by Foley et al. (2010) and others evaluated the potential of active video games to improve energy consumption and physical activity habits in children [27]. Compared to conventional passive video games, playing active video games leads to a greater energy expenditure, comparable to mild to moderate physical exercise. It is even possible to achieve the intensity level of High-intensity interval training (HIIT), which involves short bursts of high-intensity activity interspersed with brief rest periods or low-intensity exercise [28]. A recent study [29] described Exergaming as an excellent, safe, and innovative way to create enjoyable HIIT, workouts, potentially promoting sustained participation in physical activity among adolescents due to the enjoyable nature of the activity [29–31].

Although Exergaming have been around since the 1980s [18], researchers have only recently begun to pay closer attention to the potential of exergaming therapies for children and specific demographics. Most research in this field focuses on executive functions and motor abilities. Executive functions refer to cognitive processes and mental skills responsible for managing and regulating various complex cognitive tasks [32]. These functions encompass three primary areas: inhibitory control, working memory, and cognitive flexibility [33, 34]. Motor skills, on the other hand, refer to the ability and coordination of muscles and limbs to perform movements and actions. They are categorized into locomotor skills (like sprinting and jumping), manipulative skills (like throwing and catching), and stability skills (like pivoting and balancing) [35]. Executive functions and motor skills play a vital role in children’s social, cognitive, and physical development and are thought to lay the groundwork for an active lifestyle [36, 37].

Several studies have shown positive impacts of Exergaming on physical activity levels, motor skills, and body composition in children with intellectual impairments [38]. Similarly, Exergaming has been suggested to improve motor coordination in survivors of childhood brain tumors [39]. Previous reviews analyzing the use of exergames in children with Autism Spectrum Disorder (ASD) suggest that exergames can be a potential therapeutic tool. These games may improve physical health and cognitive functioning and reduce repetitive behaviors in children and adolescents with ASD [40, 41]. Another study using Exergaming on the Xbox Kinect with children with ADHD demonstrated substantial gains in both motor abilities and executive function [42]. For obese children, Exergaming appears to be an effective way to combat childhood obesity [43–45]. It may also benefit the psychological and mental well-being of overweight or obese children and adolescents. However, the effects on motor functions in this population remain unclear [43]. Research using meta-analyses has revealed broader benefits of Exergaming. Studies have shown that Exergaming can improve executive function in patients with neurological disorders [46] and enhance inhibitory control, cognitive flexibility, and working memory in children [47]. Additionally, Exergaming is indeed effective for enhancing specific aspects of health fitness and motor skills in adolescents and children of healthy weight [48, 49]. Nevertheless, further investigation is essential to specifically assess its effects on exercise capacity. One study found no significant difference between Exergaming and traditional sports in enhancing executive function in healthy children and adolescents [50], which could be attributed to the fact that the included studies only involved healthy participants and did not include children with pre-existing executive function disorders.

Combining the findings from previous research reveals a knowledge gap. While meta-analyses have been conducted, they have not addressed how Exergaming’s effects vary across different groups of children. Additionally, no prior study has comprehensively examined the impact of motor play on both executive function and motor abilities in children. Given the widespread adoption and rapid growth of exergames, a more thorough exploration of their potential benefits is warranted. This could significantly contribute to promoting children’s health, with positive implications for public health and education. To address this knowledge gap, we conducted a systematic review and meta-analysis. Our research focused on the effectiveness of Exergaming interventions in enhancing executive function and motor abilities in children and adolescents. We specifically aimed to evaluate how well Exergaming improves these cognitive and motor skills across different groups of children.

## Materials and methods

### Protocol

This study adhered to the most recent Preferred Reporting Items for Systematic Reviews and Meta-Analyses (PRISMA-2020) reporting guidelines [51]. The checklist can be found in **S1 Checklist**. The protocol was prospectively registered with PROSPERO (CRD42023482281) and is available at https://www.crd.york.ac.uk/PROSPERO/display_record.php?RecordID=482281. **S3 File** also contains the protocol details.

### Search strategy

We searched several English databases, including Web of Science, Embase, PubMed, SPORT Discus, Cochrane Central Register of Controlled Trials, PsycINFO, and Scopus, from inception to November 25, 2023, for randomized controlled trial (RCTs) investigating the effects of Exergaming on children’s executive functions and motor ability. To ensure comprehensiveness, we also manually searched the references of the included studies. Details of the search strategy can be found in **S1 Table**.

### Criteria for inclusion and removal

This study included RCTs that investigated the effects of Exergaming interventions on executive function and motor abilities in children and adolescents under 18 years old. Included interventions were physically engaging Exergaming or Active Video Game programs, with no restrictions on frequency, intensity, duration, or type. Comparators were controlled and could include no intervention, waiting list placement, conventional exercise, or video watching. Outcomes of interest were executive functions or motor abilities measured by any validated scales or tests. Studies were excluded if they involved: (a) video games that did not require physical activity, (b) non-interventional designs such as protocol reviews, cohort studies, case-control studies, book chapters, or conference articles, or (c) studies with missing or incomplete data.

### Study selection and data extraction

Following our defined search strategy and inclusion/exclusion criteria, two independent reviewers assessed the titles and abstracts of all retrieved studies. Full papers meeting the initial criteria then underwent a thorough review process. Two authors independently extracted data from these papers, and any discrepancies were resolved through consultation with a third author until a consensus was reached. The data extraction form captured details on both study and sample characteristics, including author names, publication year, country, study design, sample size, age distribution, sex ratio, intervention type, frequency, intensity, duration per session, total intervention duration, control information, and all relevant study outcomes.

### Study quality assessment and quality of evidence

To minimize bias, two researchers independently assessed the risk of bias in each study using the Cochrane Risk of Bias 2 tool [52]. This tool evaluates five key domains: the randomization process, deviations from intended interventions, missing outcome data, outcome measures employed, and selection of reported outcomes. Any disagreements between the researchers were resolved through discussion. Each study was then categorized as having a low, high, or unclear risk of bias based on the tool’s criteria.

The Grading of Recommendations, Assessment, Development, and Evaluation (GRADE) approach was employed to assess the certainty of evidence for each outcome, considering five downgrading factors: risk of bias, inconsistency, indirectness, imprecision, and publication bias [53]. This resulted in the categorization of evidence quality into four levels: very low, low, moderate, and high.

### Statistical analysis

This study utilized Stata 17.0 and Review Manager 5.3 software to analyze the dat. Standardized mean difference (SMD) served as the effect size metric, accompanied by a 95% confidence interval (CI). To calculate SMD, the standard deviation (SD) post-intervention was divided by the mean difference between pre- and post-intervention changes [54]. Statistical significance was established at p < 0.05. Furthermore, the inverse-variance method was employed to weigh the effect size. This method allocates greater weight to studies with more precise estimates, indicated by lower variance. In essence, the weight (Wi) for each study was calculated as Wi = 1/Vi, where Vi represents the variance of the effect estimate from that particular study.

To account for the statistical heterogeneity assessed by the chi-square test and I^2^ value, and the clinical heterogeneity due to our inclusion of children with various disorders alongside healthy children, we employed a random effects model for all meta-analyses [55]. Additionally, we performed sensitivity analyses (excluding trials one by one when I^2^ > 50%) and subgroup analyses to explore potential sources of heterogeneity. Finally, we assessed for publication bias using Egger’s test and constructed funnel plots using Stata 17.0 software.

## Results

### Selection process

**Fig 1:**
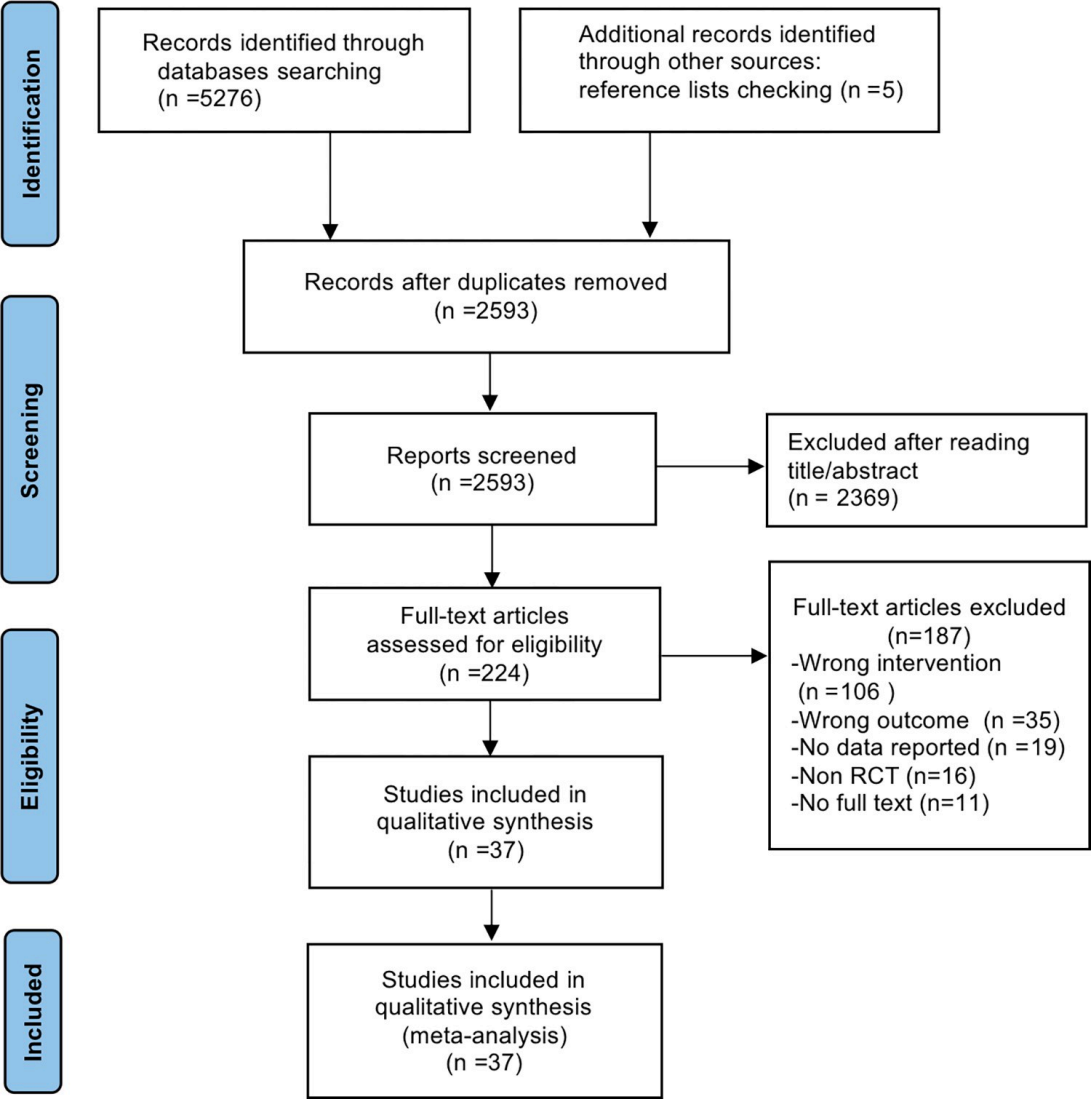
PRISMA flow diagram of the study selection.

A systematic search strategy identified 5276 articles. Following the removal of duplicates (resulting in 2593 articles), we applied pre-defined inclusion and exclusion criteria to the remaining pool. This process yielded 37 RCTs for final inclusion in the meta-analysis. The detailed search and selection process is further illustrated in Fig 1.

### Basic characteristics of included studies

This study employed a sample of 2,771 children and adolescents aged 3 to 18 years. Participants were divided into two groups: a control group (n = 1388) and an experimental group (n = 1383). The experimental group engaged in exercise game interventions, while the control group participated in daily physical activities. Refer to Table 1 for further details on the specific interventions in each group.

**Table 1:** Summarizes the characteristics of the included studies in the meta-analysis.

Reference	Disease diagnosis	Participant characteristics	Intervention characteristics	Outcome measures
Nekar et al., [56] 2022 (Korea)	ASD	Experimental(n = 12):6-18y, 10 males. control (n = 12):6-18y;12males.	Kinect: 15 minutes twice a week for 4 weeks.	Cognitive flexibility: (WCST) Cognitive inhibition: (Stroop)
Dickinson et al., [57] 2014 (UK)	ASD	Experimental (n = 50):33 under 11y;39 males. Control(n = 50):34 under 11y; 40 males.	Nintendo Will:3 weekly 15-min classes for 1 year.	Cardiopulmonary:(Bleep Test)
Milajerdi et al., [58] 2020 (Iranian)	ASD	Experimental(n = 20):8.15±1.50y;19 males. control group(n = 20):8.45±1.43y;19 males.	Xbox Kinect: 3 weekly 15-min classes for 8 weeks	Executive Function: (WCST) Motor Skill: (MABC-2)
Benzing(1) et al., [59] 2018(Switzerland)	ADHD	Experimental(n = 24): 18-12y;20 males. Control (n = 22): 18-12y;18males.	XBOX Kinect: 15-min classes.	Cognitive inhibition, cognitive flexibility: (Flanker Task)
Benzing(2) et al., [42] 2019(Switzerland)	ADHD	Experimental(n = 28): 10.46±1.30y;24 males. Control (n = 23): 10.39±1.44y;19 male.	XBOX Kinect: 3 weekly 30-min classes for 8 weeks.	Switching: (Flanker task) Inhibition: (Simon Task)
Chang et al., [60] 2022(China)	ADHD	Experimental(n = 16): 8.38±1.20y;13 males. Control (n = 16): 8.38±1.31y;13 males.	Nintendo Will:3 weekly 60-min classes for 12 weeks.	Executive Function: (WCST)
Adamo et al., [61] 2010(Canada)	Obese	Experimental(n = 13): 13.9±1.4y;7 males. Control (n = 13): 15.1±1.8y;7 male.	Game Bike: 2 weekly 60-min classes for 10 weeks.	Cardiopulmonary: (Peak workload)
Staiano et al., [62] 2012(America)	Obese	Experimental(n = 27):15-19y, 12males. Control (n = 27):15-19y;11male.	Wii EA Sports: 1 weekly 30-min classes for 10 weeks.	Executive Function:(D-KEFS)
Alsaif et al., [63] 2015 (Saudi)	CP	Experimental(n = 20):6-10y, 10 males. Control (n = 20):6-10y;12male.	Nintendo Wii Fit: 7 weekly 20-min classes for 12 weeks.	Motor performance: (MABC-2); Balance: (BOTMP 5:6)
Bonney et al., [64] 2017 (Africa)	DCD	Experimental(n = 21):13-16y;21 females. Control (n = 22):13-16y;22 females.	Nintendo Wii:45-min classes weekly for 14 weeks.	Motor performance: (MABC-2)
Chen (1) et al., [65] 2013 (China)	CP	Experimental(n = 12):3-5y;10 males. Control (n = 12):3-5y;12male.	Q4 Scene Interactive Training System: 5 weekly 30-min classes for 12 weeks.	Balance: (BBS); Motor performance: (GMFM-88)
Chen (2) et al., [66] 2016 (China)	CP	Experimental(n = 20):3-5y;10 males. Control (n = 20):3-5y;8 male.	Q4 Scene Interactive Training System: 5 weekly 40-min classes for 12 weeks.	Balance: (BBS); Motor performance: (GMFM-88)
Chiu et al., [67] 2014 (China)	CP	Experimental(n = 30):6-13y. Control (n = 27):6-13y.	Wii Sports: 3 weekly 40-min classes for 6 weeks.	Hand function: (Nine hole Peg Test)
Chunhee et al., [68] 2016(Korea)	CP	Experimental(n = 9):9-10y. Control (n = 9):9-10y.	Nintendo Wii: 3 weekly 30-min classes for 8 weeks.	Motor performance:(GMFM) Balance: (PBS)
Mombarg et al., [69] 2013 Netherlands	Healthy	Experimental(n = 15):8-11y;12 males Control (n = 14):8-11y;11 male.	Nintendo Wii Fit: 3 weekly 30-min classes for 6 weeks.	Motor performance: (MABC-2); Balance: (BOT-2)
Cavalcante Neto et al., [70] 2018 Brazil	DCD	Experimental(n = 16):7-10y. Control (n = 16):7-10y.	Nintendo Wii: 2 weekly 60-min classes for 8 weeks.	Motor Skill: (MABC-2)
Ren et al., [71] 2016(China)	CP	Experimental(n = 19):6-18y;10 males Control (n = 16):6-18y;10males.	Q4 Scene Interactive Training System: 5 weekly 40-min classes for 12 weeks.	Balance: (BBS); Motor performance: (GMFM-88)
Sxahin et al., [72] 2020 (Turkey)	CP	Experimental(n = 30): 10.5 ± 3.62y;17 males. Control (n = 30): 10.06 ± 3.24y; 20 males.	XBOX Kinect: 2 weekly 45-min classes for 8 weeks.	Motor performance: (BOTMP-SF)
Tarakci et al., [73] 2016 (Turkey)	CP	Experimental(n = 15): 10.46 ± 2.69y; 5 males. Control (n = 15): 10.53 ± 2.79y; 6 males.	Nintendo Wii: 2 weekly 50-min classes for 12 weeks.	Balance: (BBS)
Urgen et al., [74] 2016 (Turkey)	CP	Experimental(n = 15): 11.07 ± 2.37 y;7 males. Control (n = 15): 11,33 ± 2,19 y; 7 males	Nintendo Wii Fit: 2 weekly 45-min classes for 9 weeks.	Motor performance:(GMFM) Balance: (Timed-up and go)
Atasavun Uysal et al., [75] 2016 Turkey	CP	Experimental(n = 12):6-14y;8 males. Control (n = 12):6-14y;2 male.	Nintendo Wii: 2 weekly 30-min classes for 12 weeks.	Motor performance:(GMFCS)
Zhao (1) et al., [76] 2018 (China)	CP	Experimental(n = 25):3-5y;15 males. Control (n = 25):3-5y;13 male.	XBOX Kinect: 5 weekly 30-min classes for 3 weeks.	Motor performance:(GMFM)
Zhao (2) et al., [77] 2018 (China)	CP	Experimental(n = 24):3-6y;11 males. Control (n = 24):3-6y;16 males.	XBOX Kinect: 5 weekly 40-min classes for 3 weeks.	Motor performance:(GMFM)
Salem et al., [78] 2012(America)	DD	Experimental(n = 20):3-5y;12 males. Control (n = 20):3-5y;10 males.	Nintendo Wii Fit: 2 weekly for 10 weeks.	Motor performance:(GMFM)
Medeiros et al., [79] 2018 (Brazil)	Healthy	Experimental(n = 32):8-10y. Control (n = 32):8-10y.	XBOX Kinect:2 weekly 45-min classes for 9 weeks.	Motor Skill: (TGMD‑2)
McGann et al., [80] 2019 (Ireland)	Healthy	Experimental(n = 20):5-6y;11 males. Control (n = 20):5-6y;10 males.	XBOX Kinect:5 weekly 3-min classes for 8 weeks.	Motor Skill: (TGMD‑2)
Gao et al., [81] 2019 (America)	Healthy	Experimental(n = 18): 4.56 ± 0.62y;11 males Control (n = 14): 4.93 ± 0.83y;5 males.	LeapTV exergame:5 weekly 30-min classes for 12 weeks.	Cognitive Flexibility: (DCCS)
Ye et al., [82] 2019 (China)	Healthy	Experimental(n = 36):8-10y;20 males Control (n = 45):8-10y;22 males.	XBOX Kinect:1 weekly 50-min classes for 32 weeks.	Cardiorespiratory: (HMR)
Lau et al., [83] 2017 (China)	Healthy	Experimental(n = 40):8-11y;29 males Control (n = 40):8-11y;26 males.	XBOX Kinect:2 weekly 60-min classes for 12 weeks.	Cardiorespiratory: (PACER)
Sheehan et al., [84] 2013(Canada)	Healthy	Experimental(n = 21):9-10y;10 males Control (n = 21):9-10y;12 males.	Nintendo Wii Fit: 5 weekly 34-min classes for 6 weeks.	Balance: (HUR BT4™ balance platform)
Sheehan et al., [85] 2012(Canada)	Healthy	Experimental(n = 22):8-9y;12 males Control (n = 21):8-9y;8 males.	Nintendo Wii Fit: 3 weekly 34-min classes for 6 weeks.	Balance: (HUR BT4™ balance platform)
Flynn (1) et al., [86] 2018 (America)	Healthy	Experimental(n = 35):7-12y;18 males Control (n = 41): 7-12y;22 males.	Nintendo Wii Fit:20-minutes.	Cognitive flexibility: (Flanker task)
Xiong et al., [87] 2019 (China)	Healthy	Experimental(n = 30):4-5y;10males. Control (n = 30):4-5y;12males.	XBOX Kinect:5 weekly 20-min classes for 8 weeks.	Executive Functions: (DCCS)
Liu et al., [88] 2022 (China)	Healthy	Experimental(n = 24): 4.92 ± 0.28y;12 males. Control (n = 24): 4.88 ± 0.34y;11 males.	Nintendo Switch: 5 weekly 30-min classes for 4 weeks.	Executive Functions: (Early Years Toolbox)
Benzing et al., [89] 2016(Switzerland)	Healthy	Experimental(n = 21):13-16y;21 males. Control (n = 21):13-16y;21 males.	Nintendo Wii: 15 minutes.	Executive Functions: (Delis-Kaplan)
Layne et al., [90] 2020 (America)	Healthy	Experimental(n = 19):8-9y;14 males. Control (n = 21):8-9y;17 males.	Xbox Kinect: 5 weekly 10-min classes for 4 weeks.	Executive Functions: (Go/No-Go test)
Flynn (2) et al., [91] 2014 (America)	Healthy	Experimental(n = 10):10-16y;6 males Control (n = 14):10-16y;8 males.	Nintendo Wii Fit: 1 weekly 60-min classes for 4 weeks.	Executive Functions: (D‑KEFS)

ASD, Autism Spectrum Disorder; ADHD, Attention Deficit Disorder with Hyperactivity; DCD, Developmental coordination disorder; CP, Cerebral palsy; DCD, Develop-mental Delay; WCST, Wisconsin Card Sorting Test; D-KEFS, Delis-Kaplan Executive Function System; MABC, Movement Assessment Battery for Children; BBS, Berg Balance Scale; GMFM, Gross Motor Function Measure; PBS, Pediatric Balance Scale; BOT-2, Bruininks-Oseretsky Test of Motor Proficiency; BOTMP-SF, Bruininks-Oseretsky Test of Motor Proficiency, Short Form; TGMD, Test of Gross Motor Development; DCCS, Dimensional Change Card Sort; HMR, High Motor Ratio; PACER, Progressive Aerobic Cardiovascular Endurance Run.

### Risk of bias assessment

**Fig 2:**
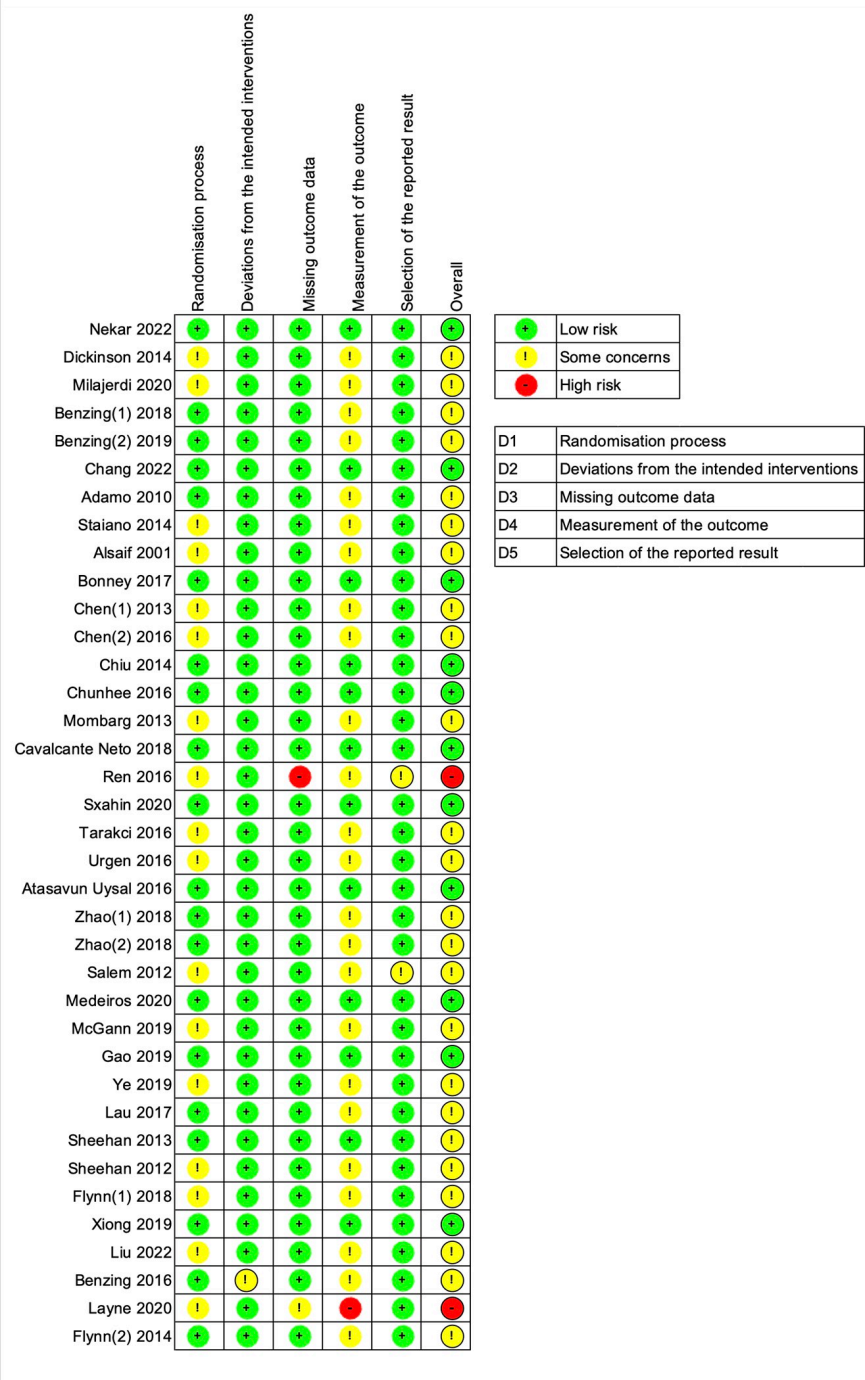
RoB2 risk of bias ratings for each eligible study.

Our assessment of bias risk across the included studies revealed that 2 studies (5.4%) had a high risk of bias, 24 studies (64.9%) had a moderate risk of bias, and 11 studies (29.7%) had a low risk of bias. The primary factor influencing this risk assessment was the potential for bias in outcome measures. As is common in exercise intervention trials, blinding participants and researchers can be challenging, potentially affecting the reliability of the measurements. A more detailed breakdown of the risk of bias assessment is provided in Fig 2.

### Quality of evidence

Due to limitations in blinding participants and outcome assessors in the included randomized controlled trials, we downgraded the overall quality of the evidence by one level. This grading system categorizes evidence quality from very low to moderate, with each outcome receiving a specific rating as detailed in **S2 Table**.

### Effect on executive functioning

#### Cognitive flexibility

**Fig 3:**
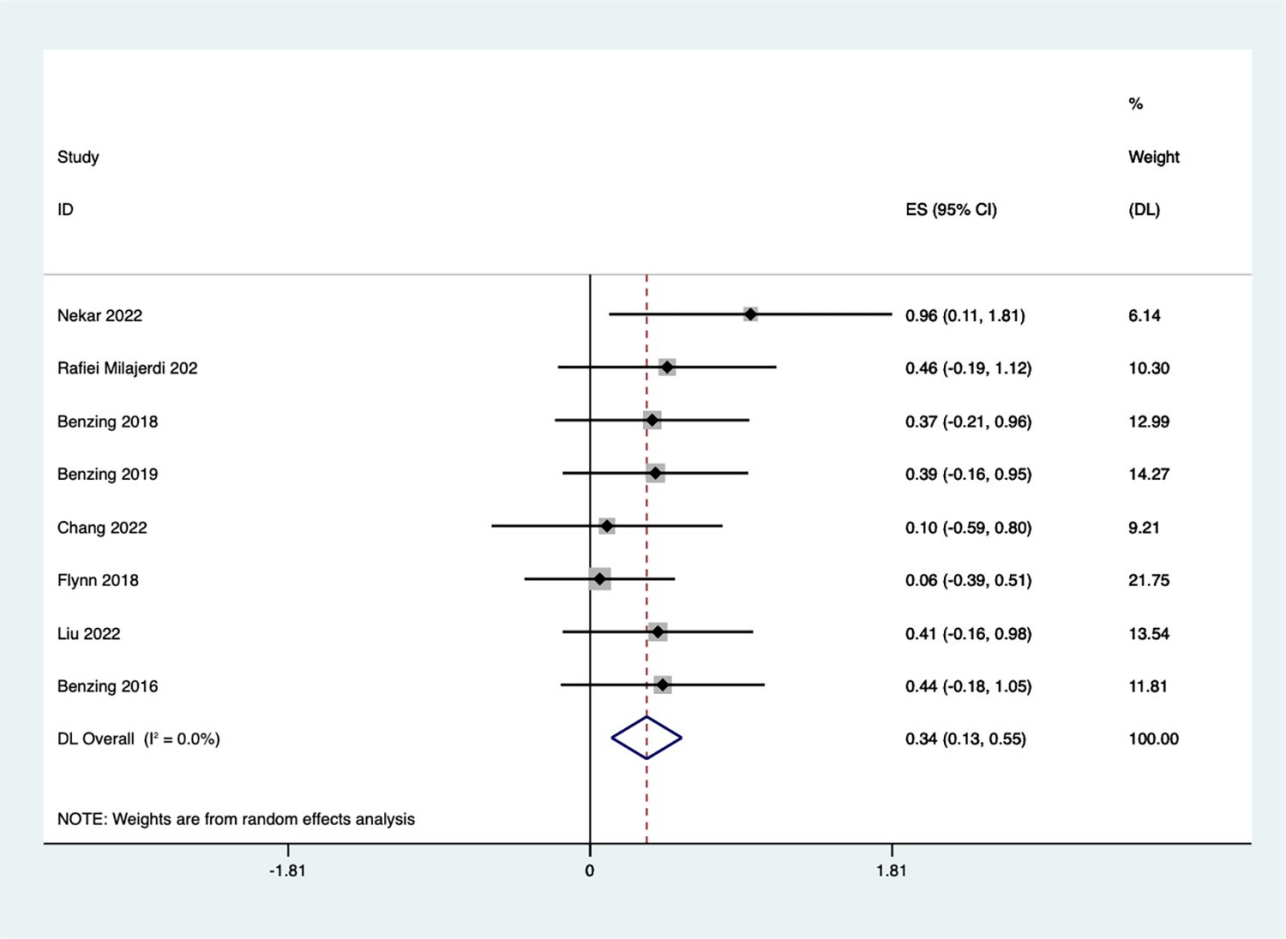
Forest plot of cognitive flexibility.

Eight studies examined the influence of exergaming on cognitive flexibility in children. The finding revealed a positive of Exergaming on children’s cognitive flexibility (Fig 3). Compared to control groups, the exercise game group exhibited a significantly higher score on the flexibility measure [SMD = 0.34, 95%CI (0.13, 0.55)]. Additionally, there was no significant heterogeneity among the studies (I^2^ = 0.0%, *P* = 0.738), suggesting consistent results across the investigations.

**Fig 4:**
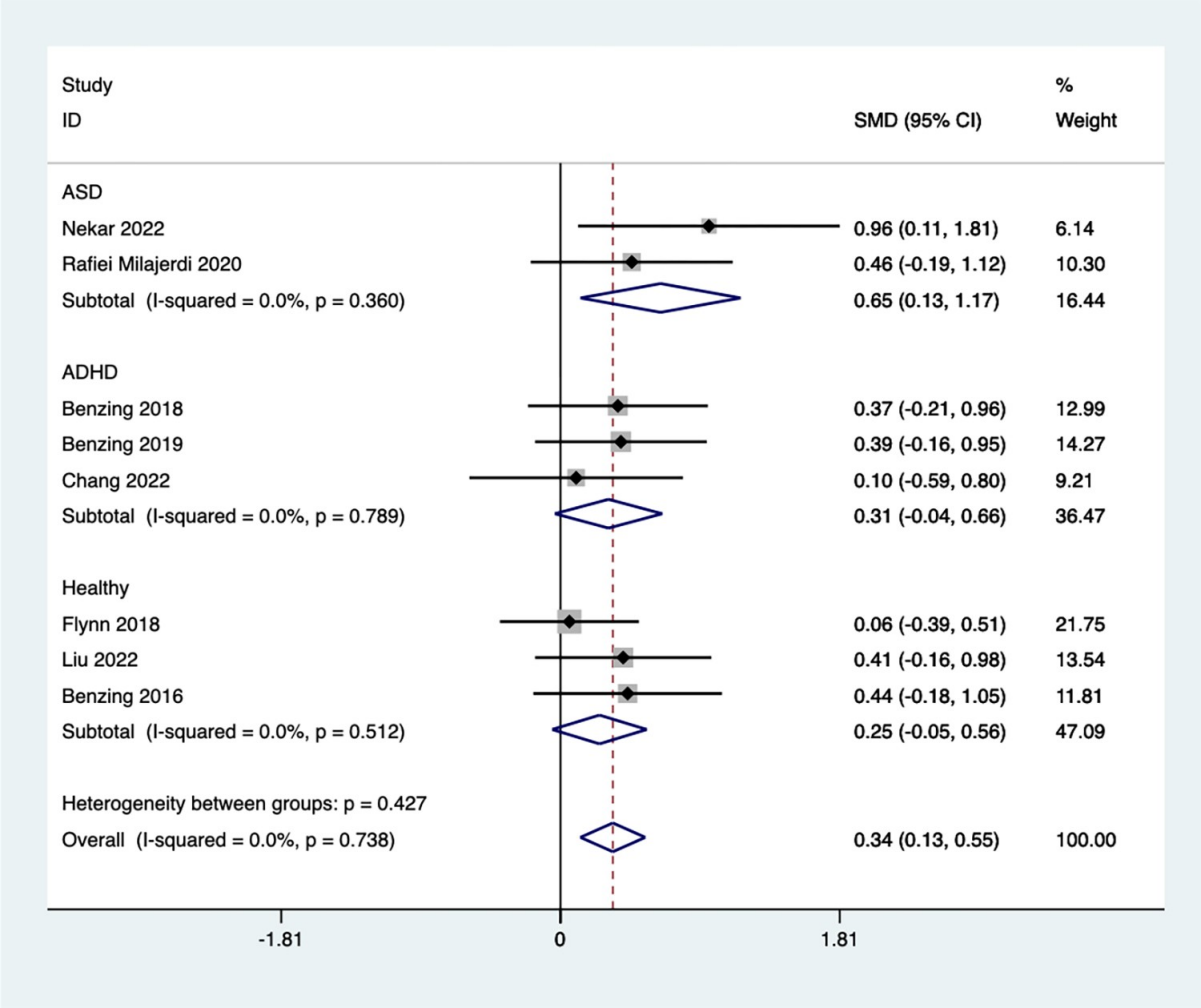
Forest plot of cognitive flexibility for group subgroup analyses.

Subgroup analysis by child population revealed significant improvements in cognitive flexibility for children with ASD [SMD = 0.65, 95%CI (0.13, 1.17), I^2^ = 0.0%, *P* = 0.36] (Fig 4). However, Exergaming did not have a significant effect on cognitive flexibility in children with ADHD [SMD = 0.31, 95%CI (-0.04, 0.66), I^2^ = 0.0%, *P* = 0.789] or healthy children [SMD = 0.25, 95%CI (-0.05, 0.56), I^2^ = 0.0%, *P* = 0.512].

This study evaluated the possibility of publication bias using a funnel plot and Egger’s test **(**S1 Fig in **S1 File)**. The Egger’s test statistic (*p* = 0.032) indicated potential publication bias in the eight included studies. the results remained robust after applying the trim and fill method **(**S2 Fig in **S1 File)**.

#### Inhibition control

**Fig 5:**
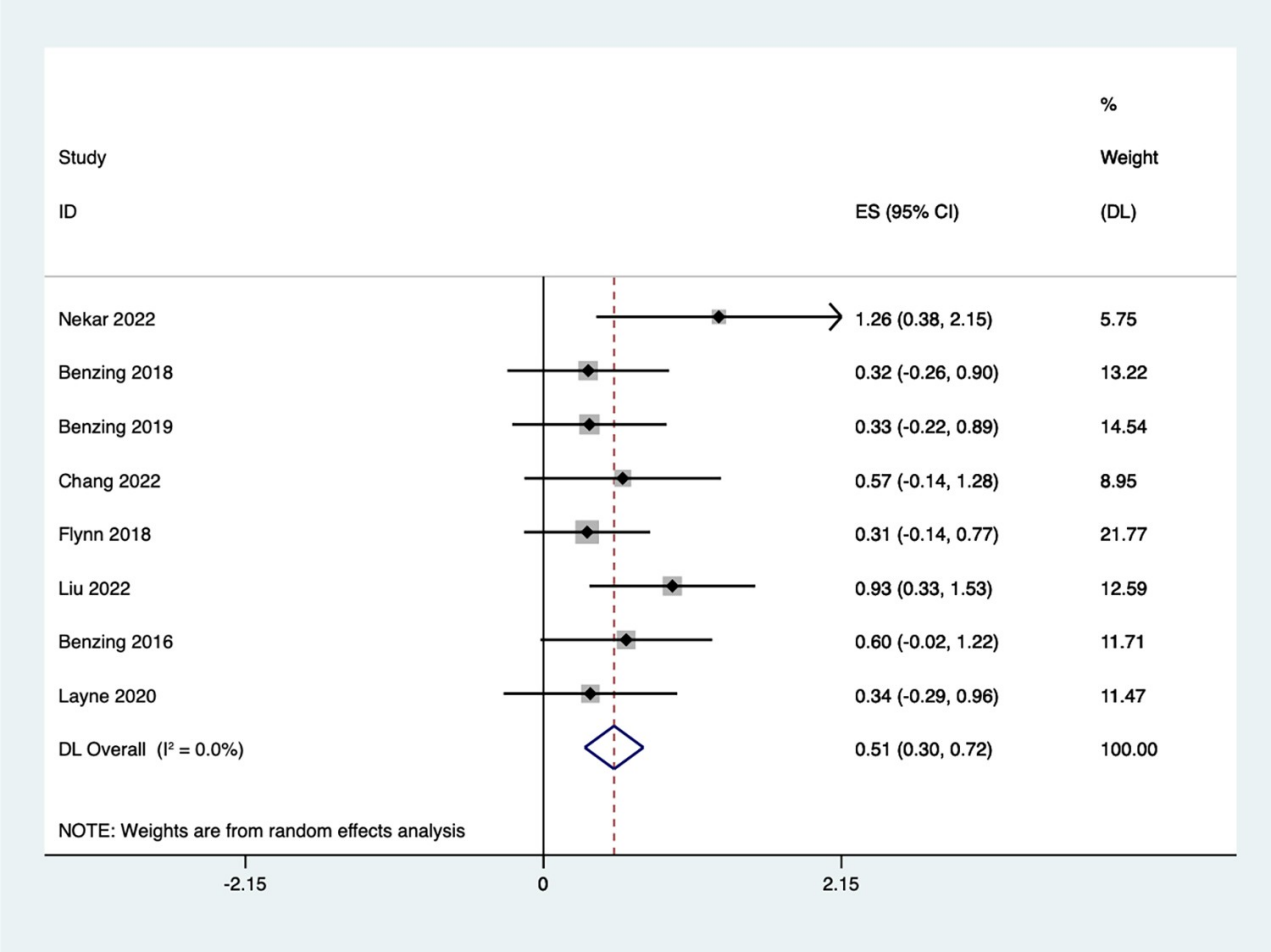
Forest plot of inhibition control.

Eight studies examined the effect of Exergaming on inhibition control (Fig 5). The meta-analysis revealed a significant improvement in the experimental group’s inhibition control score by 0.51 points compared to the control groups [SMD = 0.51, 95%CI (0.30, 0.72)]. Additionally, the analysis indicated no significant heterogeneity (I^2^ = 0.0%, *P* = 0.473).

**Fig 6:**
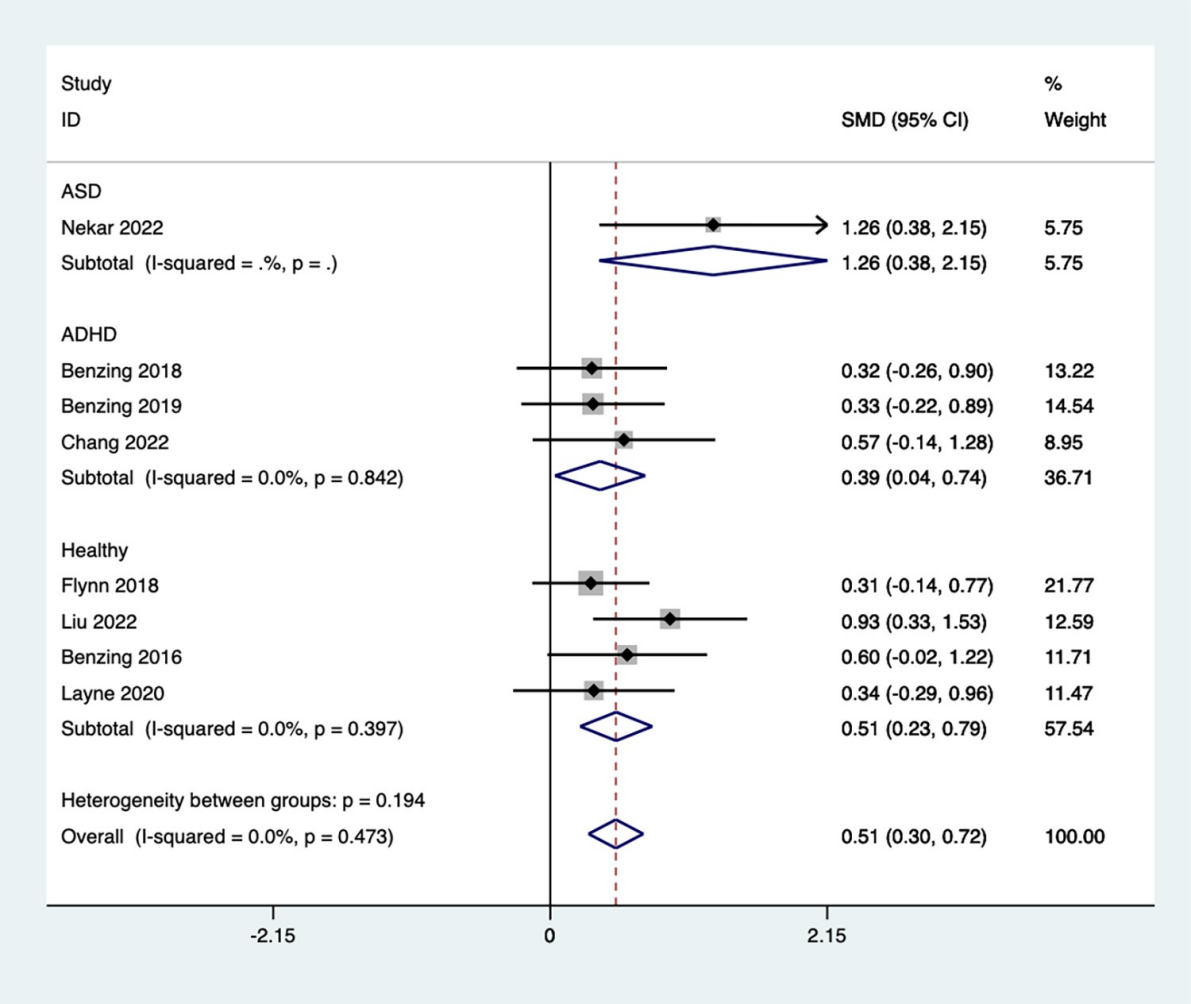
Forest plot of inhibition control for group subgroup analyses.

A subgroup analysis (Fig 6) revealed significant improvements in children’s inhibitory control function following the Exergaming intervention. This effect was observed across all groups, with children diagnosed with ASD showing the greatest improvement [SMD = 1.26, 95%CI (0.38, 2.15)]. Children with ADHD also exhibited enhanced inhibitory control [SMD = 0.39, 95%CI (0.04, 0.74), I^2^ = 0.0%, *P* = 0.842], while healthy children showed similar improvements [SMD = 0.23, 95%CI (0.23, 0.79), I^2^ = 0.0%, *P* = 0.397]. These findings suggest that Exergaming can be beneficial for improving inhibitory control.

A funnel plot was created to assess publication bias in this study **(**S3 Fig in **S1 File)**. Based on the egger shift test, p = 0.051 indicate that there is no publication bias.

#### Working memory

**Fig 7:**
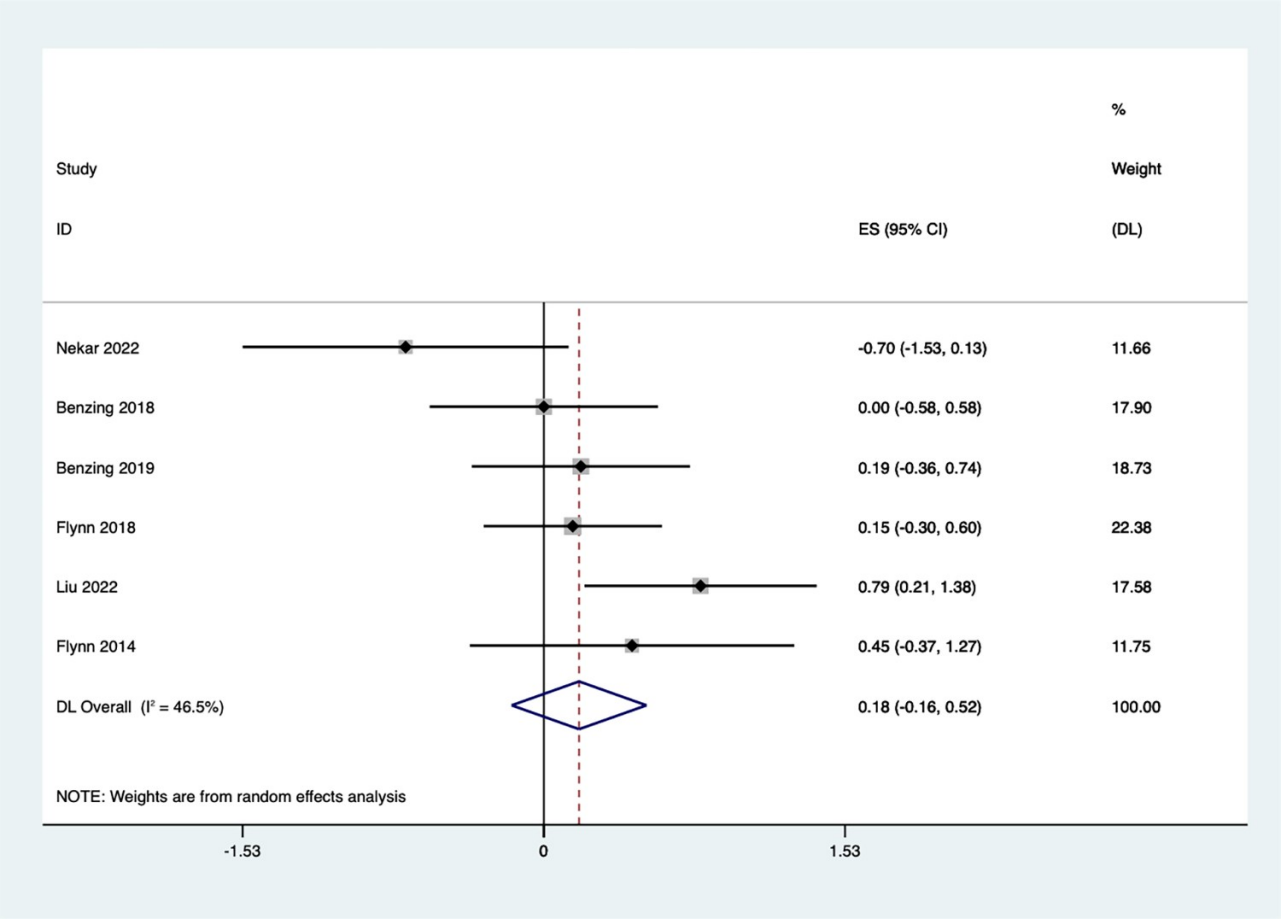
Forest plot of working memory.

Six studies examined the effect of Exergaming on working memory, using a random-effects model. The analysis revealed a non-significant effect of Exergaming on working memory [SMD = 0.18, 95% CI (-0.16, 0.52)] (Fig 7), there is no significant heterogeneity (I^2^ = 46.5%, *P* = 0.096).

**Fig 8:**
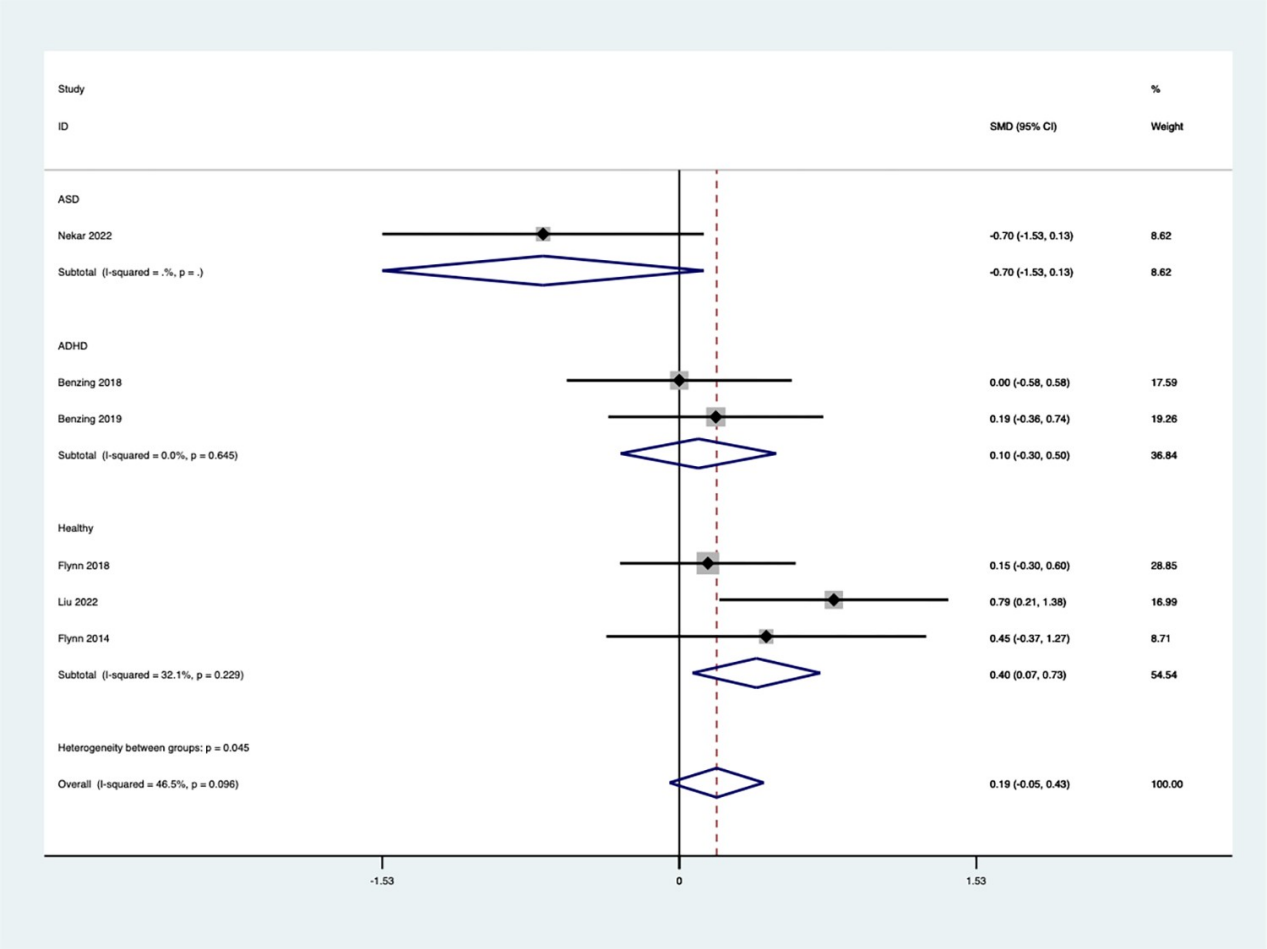
Forest plot of working memory for group subgroup analyses.

Subgroup analysis was conducted according to different children’s populations (Fig 8). The findings revealed significant variations in the effects of Exergaming across these groups. This highlights the importance of considering participant characteristics when interpreting meta-analysis results, as different populations can influence the overall conclusions. Notably, the analysis showed no significant improvement in working memory for children with ASD (SMD = -0.70, 95%CI [-1.53, 0.13]) or children with ADHD [SMD = 0.10, 95%CI (-0.30, 0.50), I^2^ = 0.0%, *P* = 0.645]. Conversely, for the subgroup of healthy children, the effect size was statistically significant [SMD = 0.40, 95%CI (0.07, 0.73), I^2^ = 32.1%, *P* = 0.229]. This suggests that Exergaming may be particularly effective in promoting improved working memory in healthy children.

To check if this study has publication bias, a funnel plot was created **(**S4 Fig in **S1 File)**. Based on the egger test, *P* = 0.655 was obtained, and the meta-analysis results showed no discernible publication bias. This further supports our confidence in the credibility and robustness of the research results and provides a more reliable basis for subsequent interpretation and application.

### Effect on global cognitive

**Fig 9:**
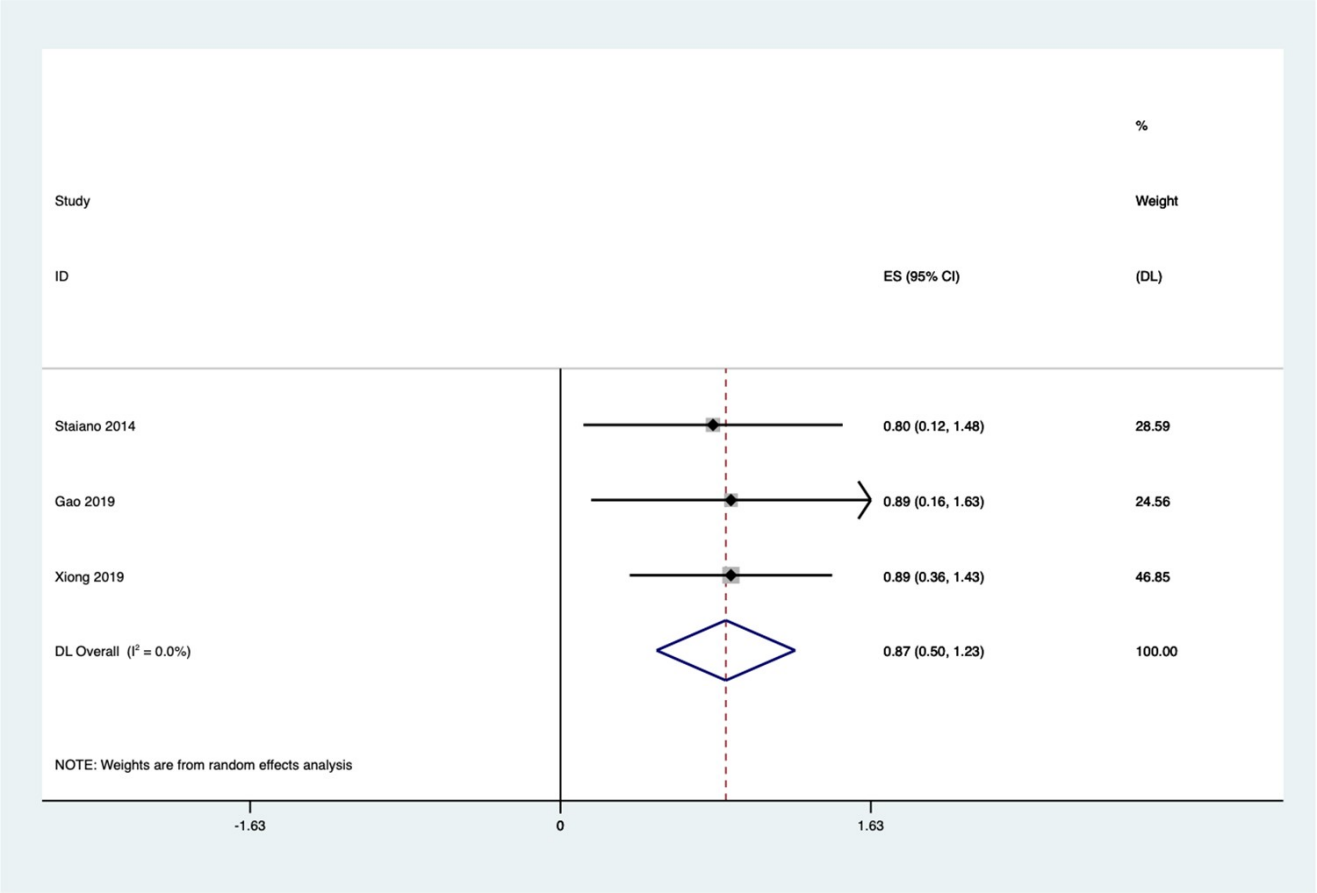
Forest plot of global cognitive.

Three studies examined the effect of Exergaming on global cognitive. The global cognitive score of the experimental group was 0.87 points higher than that of the control group, according to the results of the meta-analysis of random-effects model (Fig 9). The effect size was moderate [SMD = 0.87, 95% CI (0.50,1.23)], there is no significant heterogeneity (I^2^ = 0.0%, *P* = 0.974). Due to the limited number of studies (n = 3), assessment of publication bias and subgroup analysis were not conducted.

### Effect on athletic ability

#### Gross motor

**Fig 10:**
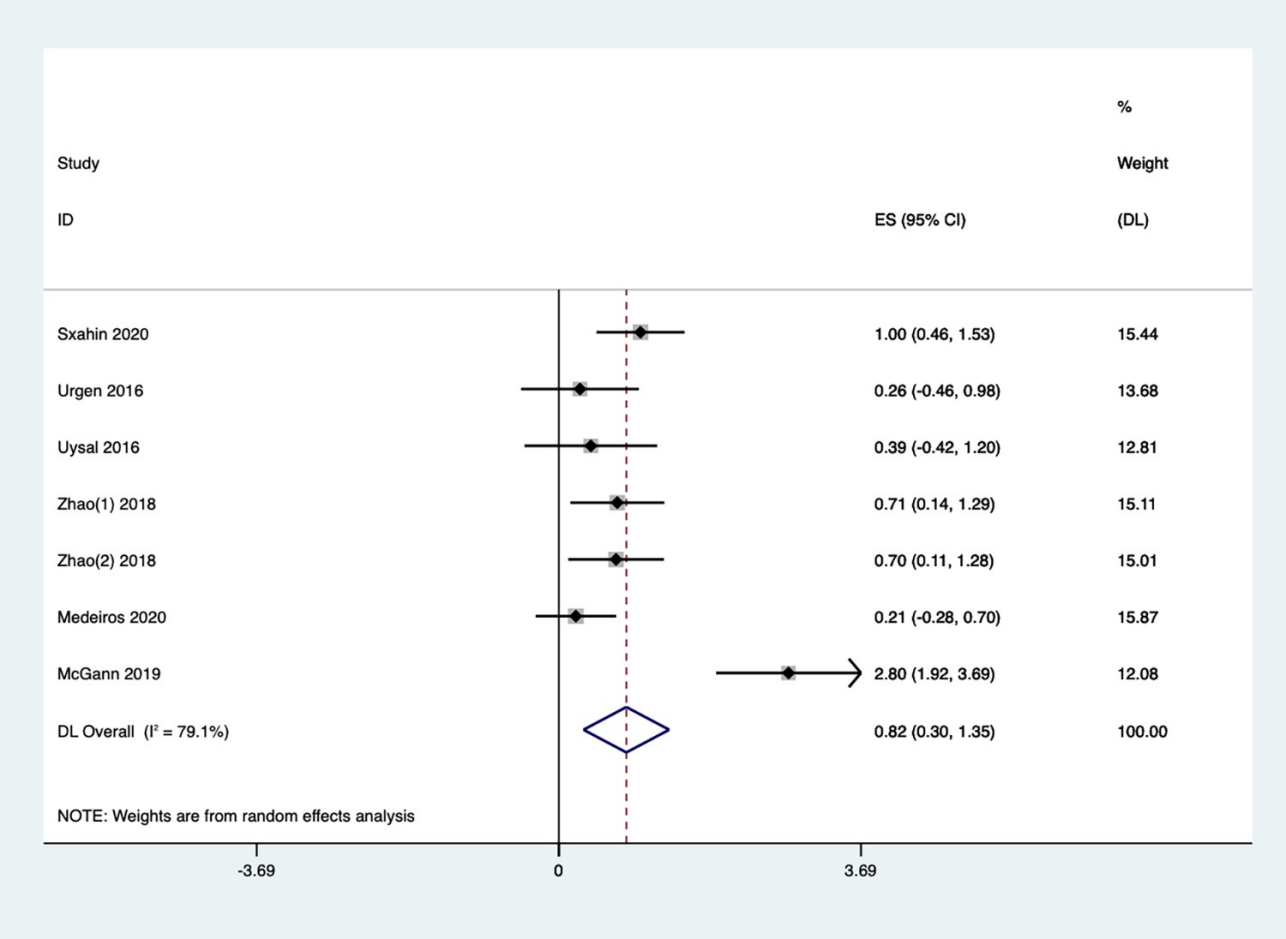
Forest plot of gross motor.

Seven studies examined the impact of Exergaming on gross motor function. Due to high heterogeneity among the studies (I^2^ = 79.1%, P < 0.1), a random-effects model was chosen for the meta-analysis. The results indicated a significant improvement (P < 0.05) in gross motor function for the Exergaming group compared to the control groups. Children in the Exergaming group scored an average of 0.82 points higher on gross motor skill assessments than the control groups [SMD = 0.82, 95% CI (0.30, 1.35)] (Fig 10).

According to the sensitivity analysis **(**S5 Fig in **S1 File)**, This analysis revealed variations in the literature depending on the participant groups examined. Notably, when two studies (Medeiros et al., 2020, Brazil [79]; McGann et al., 2019, Ireland [80]) were excluded, the heterogeneity index (I^2^) became 0%, and P-value increased to 0.531. These results suggest that the overall findings are robust **(**S6 Fig in **S1 File)**.

**Fig 11:**
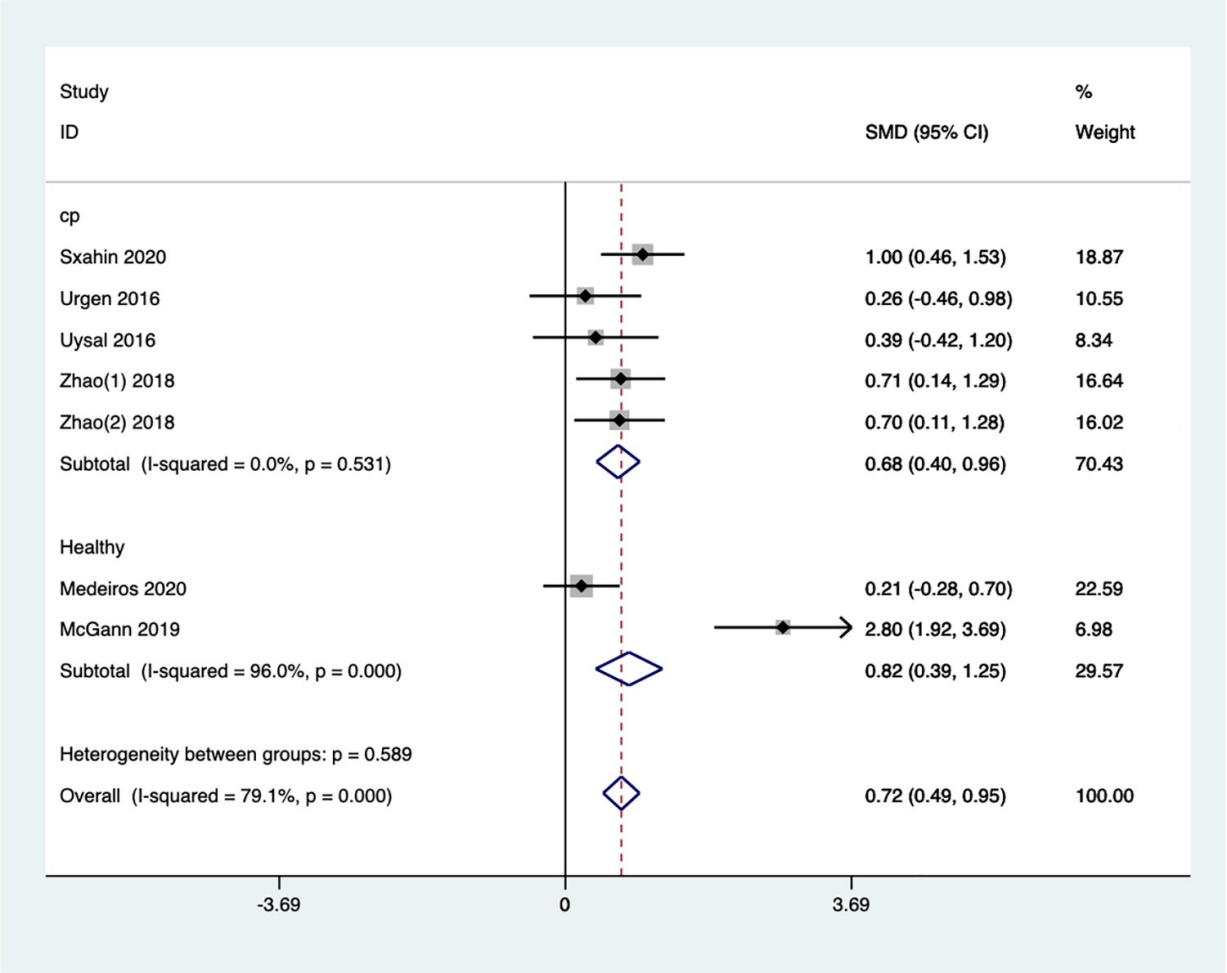
Forest plot of gross motor for group subgroup analyses.

Subgroup analysis based on different children’s groups (Fig 11) revealed significant heterogeneity in the results across populations, indicating that the effects of Exergaming may vary depending on the child’s condition. Notably, the studies on children with CP showed no heterogeneity, and the combined effect size from five studies indicated a statistically significant improvement in gross motor ability [SMD = 0.68, 95% CI (0.40, 0.96), I^2^ = 0%, *P* = 0.531]. For healthy children, the limited number of studies resulted in some heterogeneity, but the overall effect size was also significant [SMD = 0.82, 95%CI (0.39, 1.25), I^2^ = 96.0%, *P*<0.001]. These findings suggest that Exergaming can be an effective intervention for improving gross motor skills in children, particularly those with CP.

To assess publication bias, a funnel plot was generated **(**S7 Fig in **S1 File)**. The egger test yielded a P-value of 0.239, indicating no significant publication bias in this study.

#### Fine motor

**Fig 12:**
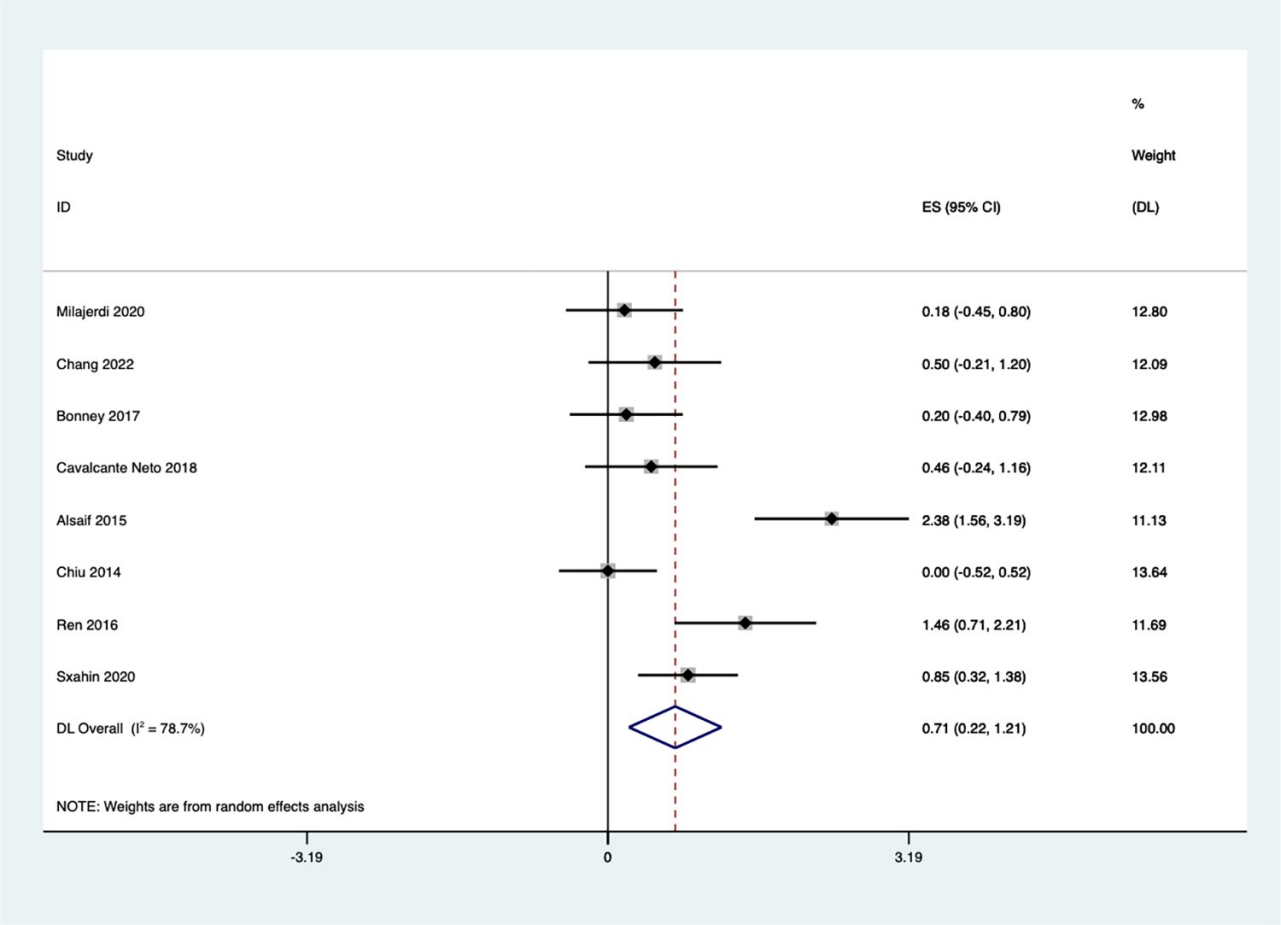
Forest plot of fine motor.

Eight studies examined the effect of Exergaming on fine motor skills. However, substantial variation was found among these studies (I^2^ = 78.7%, Q test P < 0.1), indicating significant heterogeneity. The results showed a significant improvement in the fine motor scores of the Exergaming group compared to the control groups [SMD = 0.71, 95%CI (0.22, 1.21), I^2^ = 78.7%, *P* < 0.001] (Fig 12).

A sensitivity analysis revealed that the studies’ findings may vary depending on the participants **(**S8 Fig in **S1 File)**. When excluding two studies (Alsaif et al., [63] 2015 (Saudi), and Chiu et al., [67] 2014 (China), the heterogeneity decreased (I^2^ = 47.98%, Q test P = 0.087), suggesting a more consistent effect across the remaining studies **(**S9 Fig in **S1 File)**.

**Fig 13:**
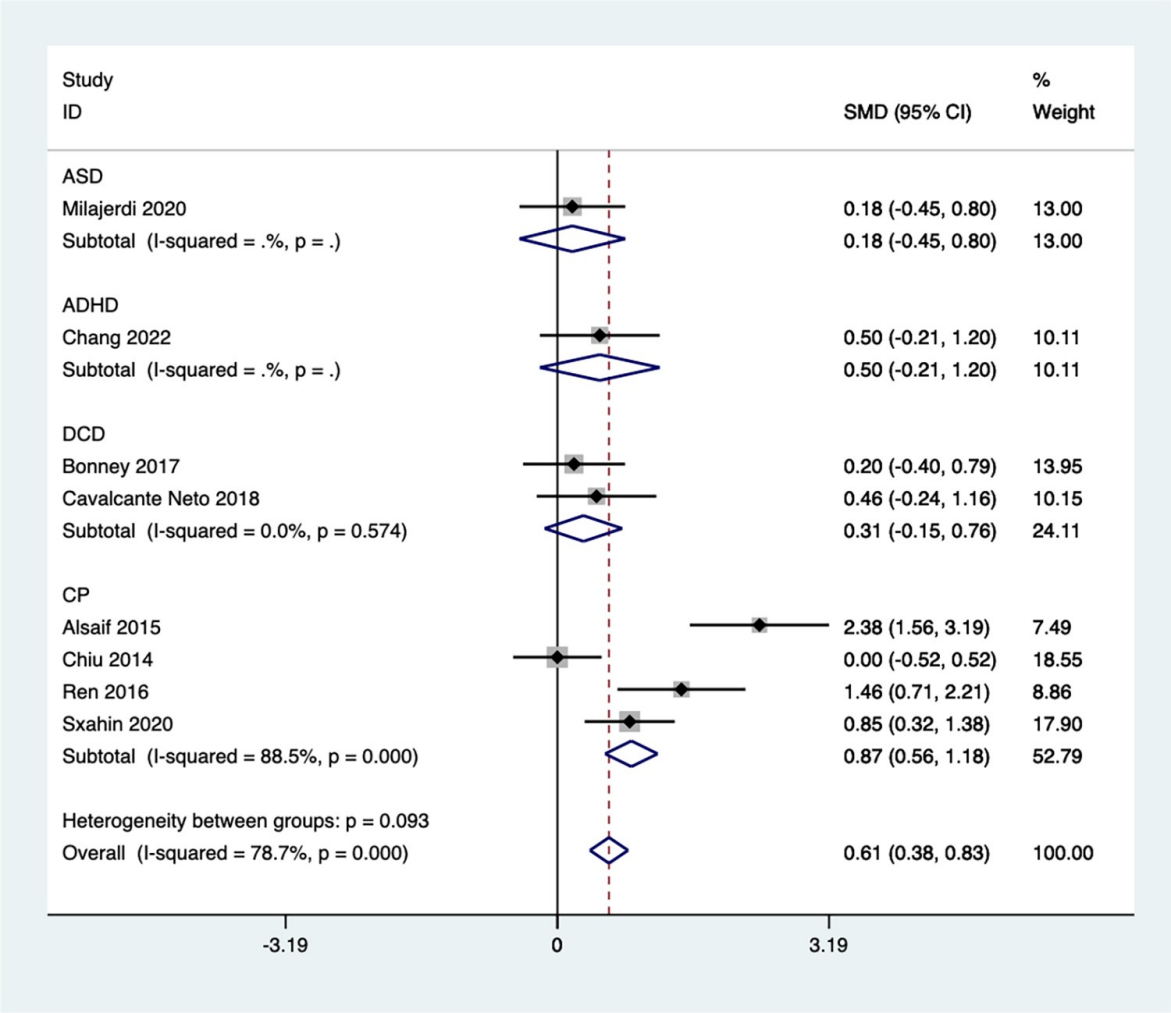
Forest plot of fine motor for group subgroup analyses.

A subgroup analysis was conducted according to different child groups (Fig 13), revealing a significant impact of group membership on the meta-analysis results. Exergaming did not lead to significant improvements in fine motor function for children with ASD [SMD = 0.18, 95%CI (-0.45, 0.80)], ADHD [SMD = 0.50, 95%CI (-0.21,1.20)], or Developmental Coordination Disorder (DCD) [SMD = 0.31, 95%CI (-0.15, 0.76), I^2^ = 0.0%, *P* = 0.574]. Conversely, the combined analysis of four studies on children with CP yielded a significant effect size (z = 5.53 *P* < 0.05), indicating that Exergaming can substantially promote fine motor abilities in this population [SMD = 0.87, 95%CI (0.56,1.18), I^2^ = 88.5%, *P* < 0.001].

A funnel plot was generated to assess for publication bias in this study **(**S10 Fig in **S1 File)**. The Egger’s regression test yielded a P-value of 0.066, indicating no statistically significant evidence of publication bias.

#### Balance

**Fig 14:**
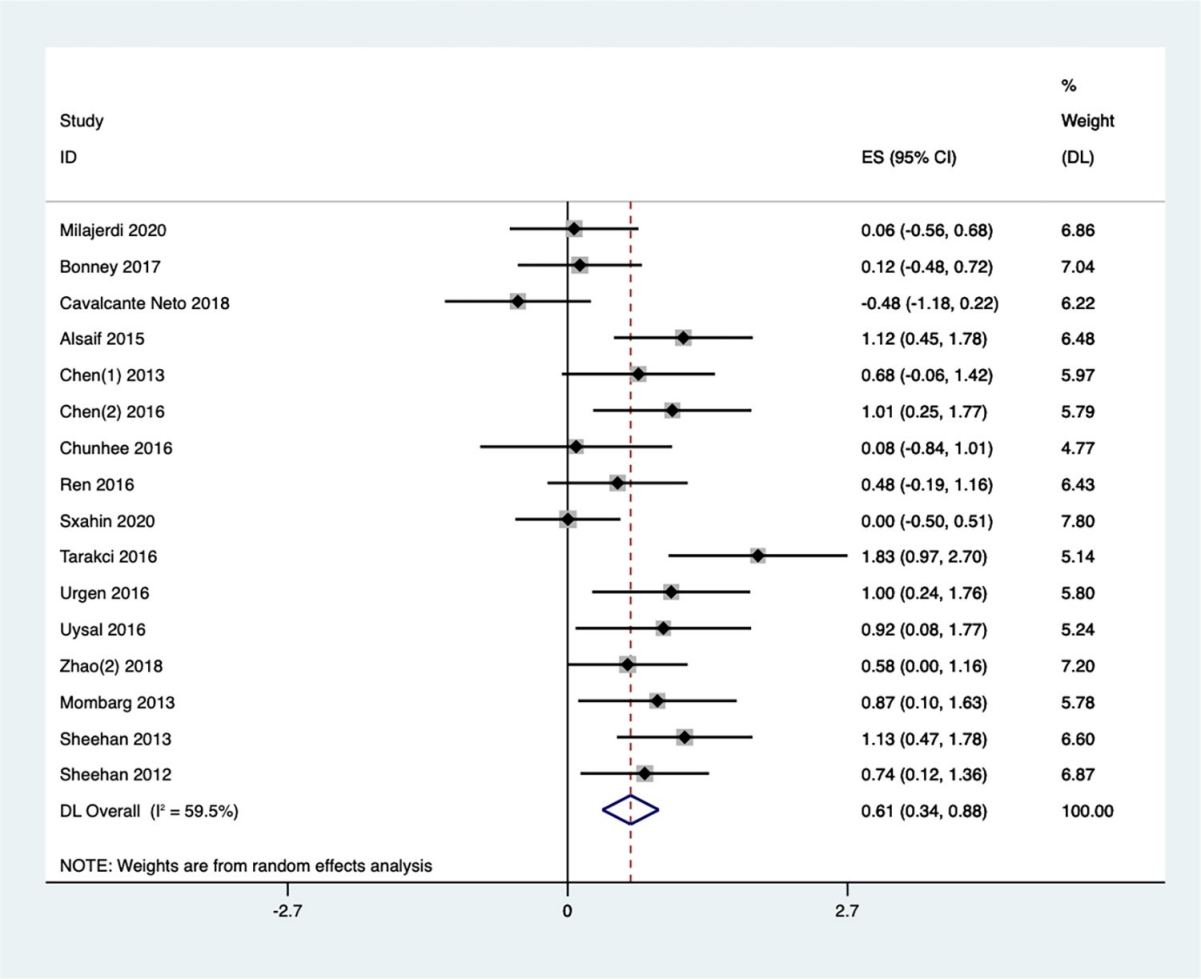
Forest plot of balance.

A meta-analysis of 16 studies investigated the impact of exergaming on children’s balance function. The analysis, based on random effects (Fig 14), showed that children in the exercise game group had statistically higher gross motor scores than those in the control group. This effect size was significant, [SMD = 0.61, 95%CI (0.34, 0.88), I^2^ = 59.5%, *P* = 0.001]. Sensitivity analysis was conducted on 16 documents this time **(**S11 Fig in **S1 File)**, and after excluding two documents: Cavalcante Neto et al., [70] 2018 (Brazil), and Tarakci et al., [73] 2016 (Turkey). This analysis resulted in a lower heterogeneity index (I2) of 35.6% (P = 0.090), suggesting moderate heterogeneity across the remaining studies **(**S12 Fig in **S1 File)**.

**Fig 15:**
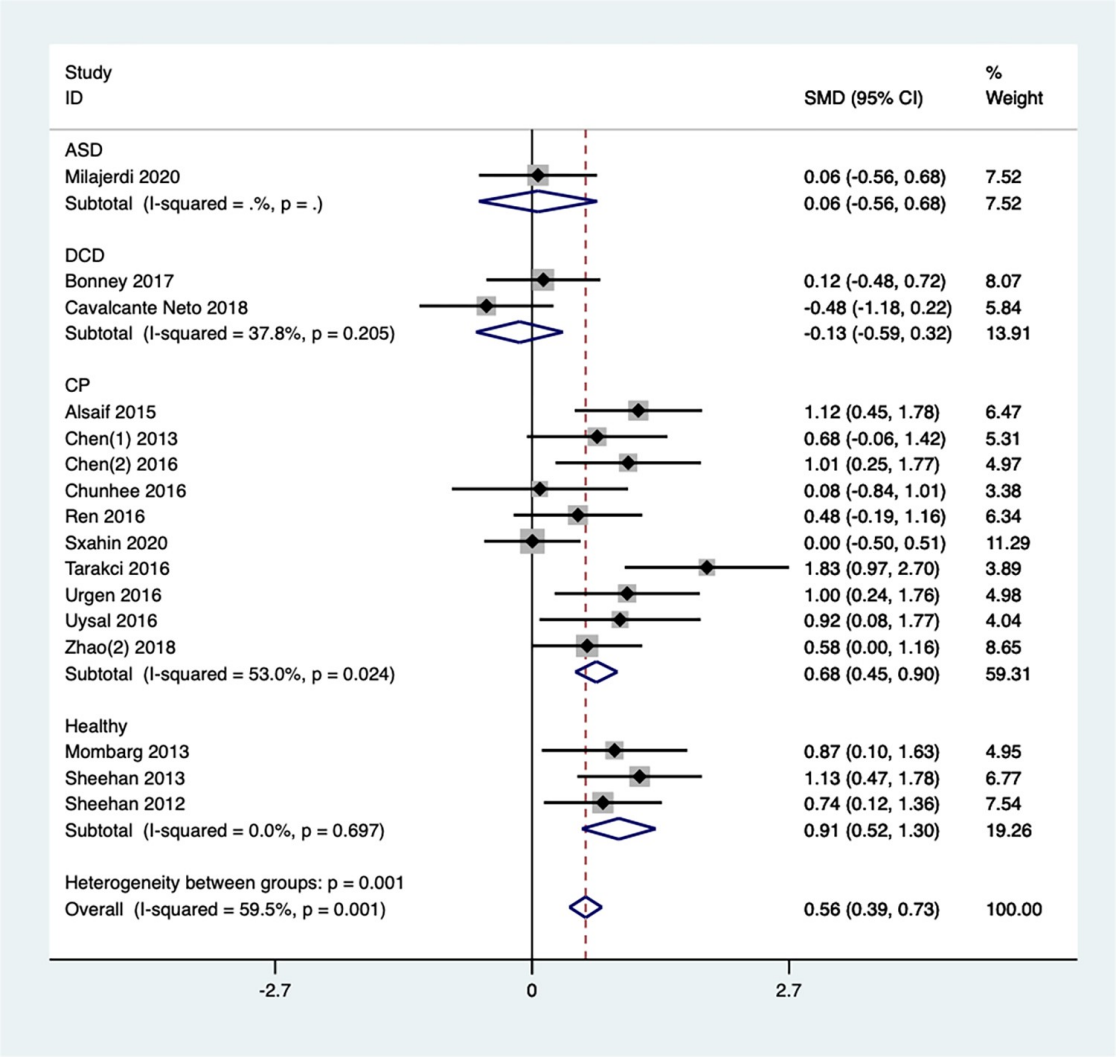
Forest plot of balance for group subgroup analyses.

We divided the 16 articles into four groups based on the participant population (Fig 15). Subgroup analysis revealed no significant improvement in balance ability for children with ASD [SMD = 0.06, 95%CI (-0.56, 0.68)] or those with DCD [SMD = -0.13, 95%CI (-0.59, 0.32), I^2^ = 37.8%, *P* = 0.205]. Conversely, combining results from 10 studies on children with CP showed a significant effect size [SMD = 0.68, 95%CI (0.45,0.90), I^2^ = 53.0%, *P* = 0.024], indicating that Exergaming can significantly improve balance in this population. Finally, pooling data from 3 studies on healthy children also yielded a significant effect size [SMD = 0.91, 95%CI (0.52, 1.30), I^2^ = 0%, *P* = 0.697], suggesting that Exergaming can substantially enhance balance ability in healthy children as well.

A funnel plot was generated to assess potential publication bias in this study **(**S13 Fig in **S1 File)**. Based on the egger shift test, p = 0.079 was obtained, it indicates that there is no publication bias.

#### Cardiorespiratory

**Fig 16:**
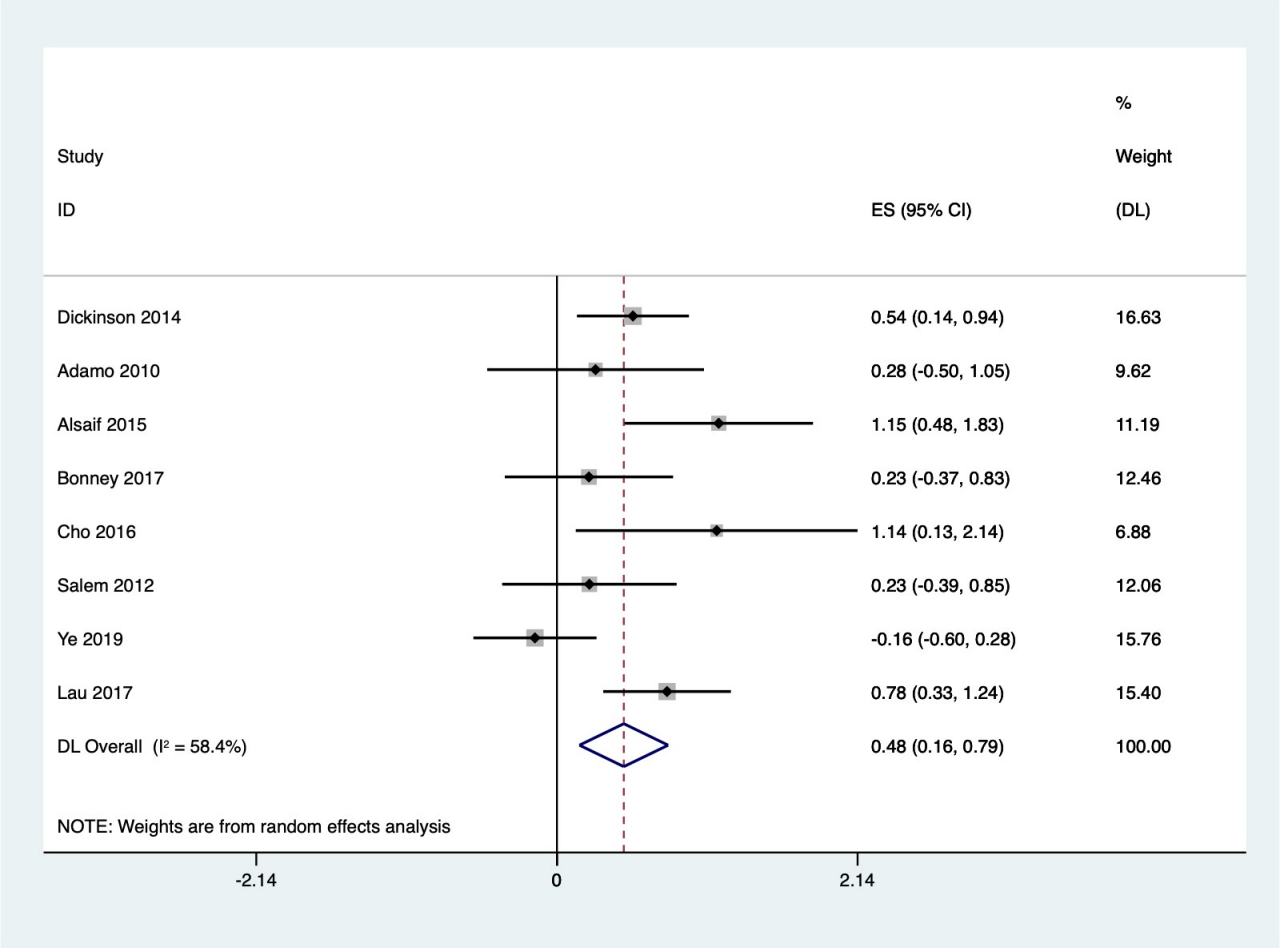
Forest plot of cardiorespiratory.

Eight studies examined the impact of Exergaming on children’s cardiopulmonary function using a random effects meta-analysis. The analysis revealed a statistically significant improvement in the Exergaming group’s cardiopulmonary function score compared to the control groups [SMD = 0.48, 95%CI (0.16, 0.79)] (Fig 16). However, there was moderate heterogeneity among the studies (I^2^ = 58.4%, *P* = 0.019). Sensitivity analysis **(**S14 Fig in **S1 File)** identified one study by Ye et al.(2019) from China [82] that significantly influenced the overall findings. After excluding this study, the heterogeneity became low (I^2^ = 24.2%, Q test *P* = 0.244), indicating robust results **(**S15 Fig in **S1 File)**.

**Fig 17:**
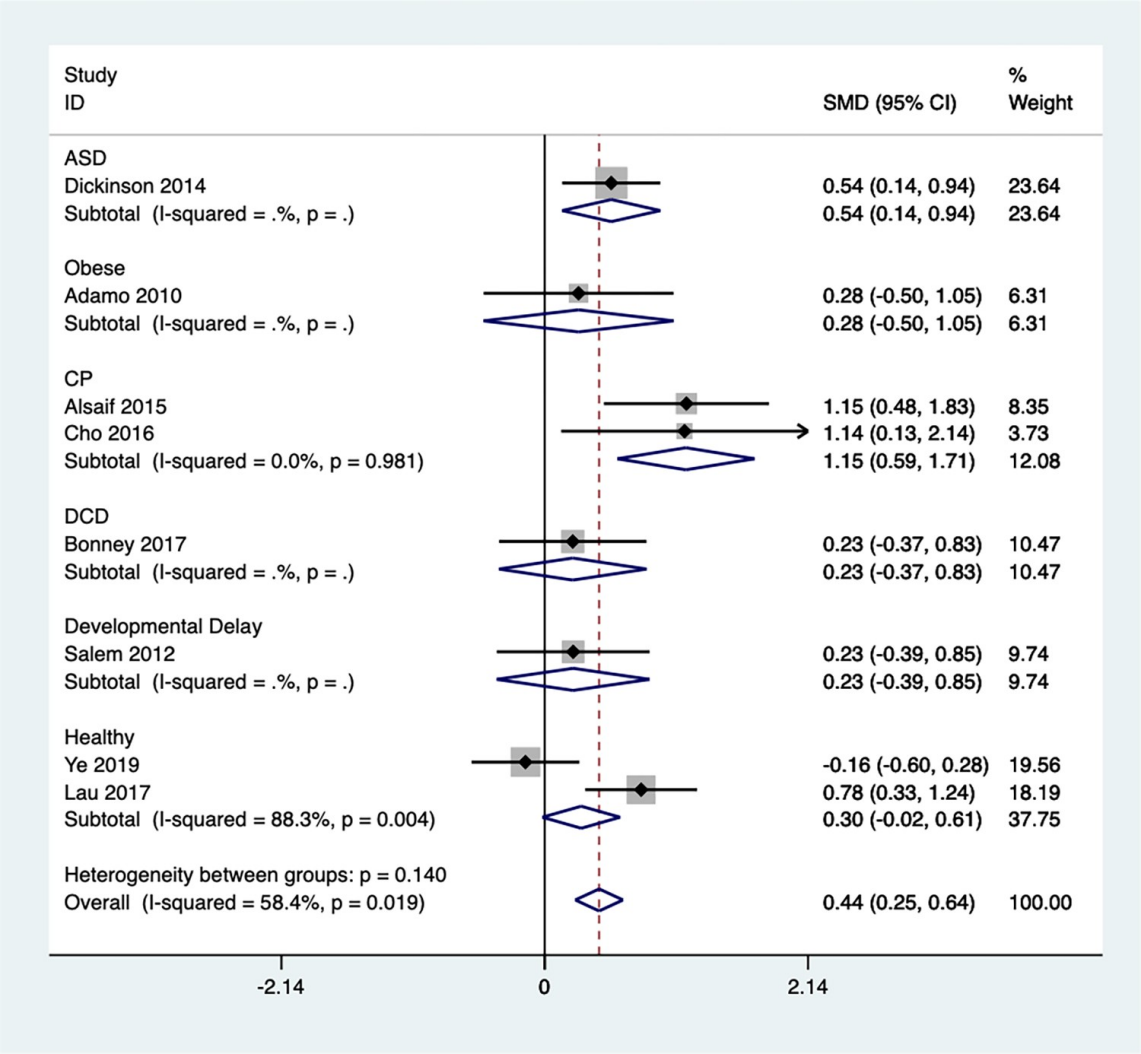
Forest plot of cardiorespiratory for group subgroup analyses.

Subgroup analysis based on children’s diagnoses (Fig 17) revealed that Exergaming significantly improved cardiopulmonary function only in children with CP [SMD = 1.15, 95%CI (0.59, 1.71)]. No significant improvements were observed in children with ASD [SMD = 0.54, 95%CI (0.14, 0.94)], obesity [SMD = 0.28, 95%CI (-0.50, 1.05)], DCD [SMD = 0.23, 95%CI (-0.37, 0.83)], or developmental delay [SMD = 0.23, 95%CI (-0.39, 0.85)]. Notably, the subgroup analysis for children with cerebral palsy showed no significant heterogeneity (I^2^ = 0.0%, *P* = 0.981), indicating a consistent effect across studies. While there was a trend towards improvement in healthy children [SMD = 0.30, 95%CI (-0.02, 0.61)], this effect was accompanied by high heterogeneity (I^2^ = 88.3%, *P* = 0.004), suggesting more research is needed to confirm this finding in this population.

A funnel plot was generated to assess potential publication bias in this study **(**S16 Fig in **S1 File)**. The Egger test results (*P* = 0.479) indicated no significant publication bias, which strengthens our confidence in the credibility and robustness of our findings. This absence of bias allows for a more reliable interpretation and application of the research outcomes.

## Discussion

This study provides a comprehensive overview of the current understanding regarding how Exergaming influences motor skills and executive functions in children and adolescents. Notably, our meta-analysis is the first to simultaneously examine the effects of exergaming on both cognitive and motor development. It accomplishes this by analyzing various motor and executive function measures across multiple randomized controlled trials involving diverse groups of children. The research demonstrates that Exergaming can positively impact children’s executive functions and motor abilities, with the extent of these benefits varying across different participant groups.

Subgroup analysis revealed that Exergaming can improve cognitive flexibility in children with ASD, mirroring the findings from the overall analysis where healthy children did not show significant improvement [47]. This aligns with the concept that individuals with lower baseline cognitive function may have a greater capacity for improvement, while those with higher initial performance may have limited room for further gains [92]. Wu et al [50] compared commercial sports games to traditional exercise programs in healthy children and found no evidence that the commercial games enhanced executive function. This could be because their study solely included healthy participants, potentially limiting the generalizability of the findings.

Physical exercise can enhance the coordinated activity between the children’s cerebellum and the prefrontal cortex in children [93]. This, along with increased neurotransmitter production, blood flow to the brain, and dopamine levels, contributes to improved cognitive function and strengthens executive function through learning and experience during exercise [94, 95]. Our findings on inhibitory control, which is most positively affected by physical activity, showed consistent improvement across all groups, aligning with previous meta-analyses [96]. This may be because the included studies focused on children and adolescents, whose inhibitory control appears more susceptible to exercise benefits compared to other aspects of executive function [97]. However, working memory improvement was only observed in the healthy children’s group, not in the ASD or ADHD groups. This could be because closed-skill activities, such as simple running or cycling, tend to yield more significant effects on working memory, which is the ability to retain information [98]. Finally, our subgroup analysis of motor abilities revealed improvements in gross motor skills and balance only for children with CP and healthy children. Fine motor skills only improved in the CP group. This might be due to enough randomized controlled trials conducted in the CP and healthy children’s groups (13 articles for CP and 14 for healthy). Similar to a previous review by Norris et al [99] that reported improved motor skills after exergaming interventions, all participants in that study were healthy children. Therefore, further research is needed to explore the effects of exergaming on motor abilities in children with multiple disorders.

Several studies have compared the effectiveness of exergames and conventional physical activities on children’s health. These studies suggest that exergames may be more beneficial for developing motor skills, particularly postural stability [84]. This advantage can be attributed to visual feedback theory. During exergaming, children can see their movements reflected on the screen in real-time, facilitating motor learning and improving balance through implicit learning [100]. Supporting this notion, a meta-analysis on children with CP found that Nintendo Wii therapy (NWT) may be a valuable tool for improving balance in this population [101]. Pope et al [102] highlighted the effectiveness of motor game interventions in promoting balance control rehabilitation in adolescents, suggesting the potential of exergames in the field of rehabilitation. Furthermore, previous research has demonstrated the broader potential of exergames for increasing physical activity in various populations [48], enhancing overall cognition compared to usual care [103] and serving as an effective treatment modality for gross motor skills in children with developmental disorders [104].

Our study has significant implications for public health and education. Exergaming offers a more accessible and enjoyable way to promote physical activity in children, potentially leading to improved performance at home and school. However, the current underestimation of exergames’ potential hinders their wider adoption. Clinically, exergames can serve as a valuable supplement to traditional therapies. Continued development of digital technology is crucial to create engaging and effective interventions specifically tailored to children’s needs.

This meta-analysis has some limitations that should be acknowledged. First, while all included studies were randomized controlled trials, blinding participants and evaluators can be challenging in exercise intervention studies. This introduces potential bias and weakens the strength of the evidence. Second, although we aimed for comprehensive literature inclusion, the number of studies analyzed for specific outcomes, particularly those related to children with ASD and ADHD, was limited. This highlights the need for further research in this area to solidify and expand upon the current findings.

## Conclusion

Our findings demonstrate that Exergaming can have a beneficial effect on children’s cognitive flexibility, inhibitory control, global cognition, and motor abilities, although the specific effects vary across different groups. Notably, no statistically significant effect was observed for working memory. Given that the current evidence ranges from very low to moderate quality, more high-quality randomized controlled trials are needed to explore the full potential benefits of Exergaming for different groups of children.

## Supporting information

S1 Checklist PRISMA checklist. (DOCX)

S1 File Appendix 1. (PDF)

S2 File Minimal data set. (XLSX)

S3 File PROSPERO protocol. (PDF)

S1 Table Searching studies. (DOCX)

S2 Table GRADE assessment. (DOCX)
