# Supplementary material for: Effects of Exergaming on executive function and motor ability in children: A systematic review and meta-analysis
Source: PLoS One. 2024 Sep 6;19(9):e0309462. doi: 10.1371/journal.pone.0309462 (PMC11379181; doi:10.1371/journal.pone.0309462)

Appendix 1

S1. Analysis for cognitive flexibility.

Figure S1. Funnel plot of cognitive flexibility.

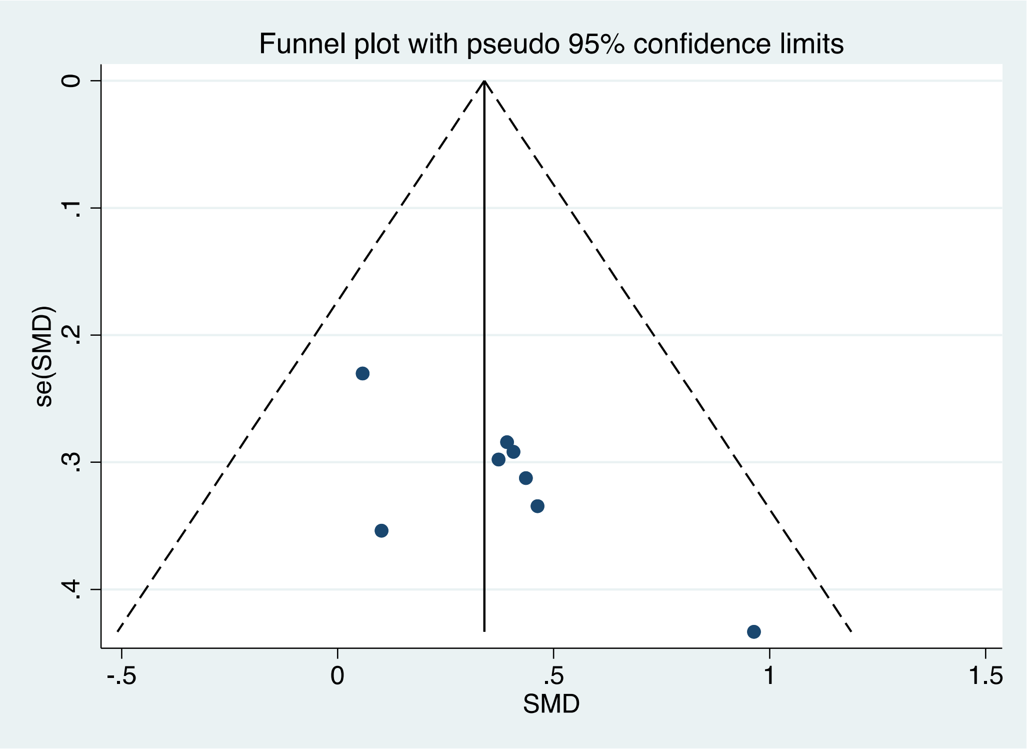

Figure S2. Funnel plot based on the trimming method (cognitive flexibility).

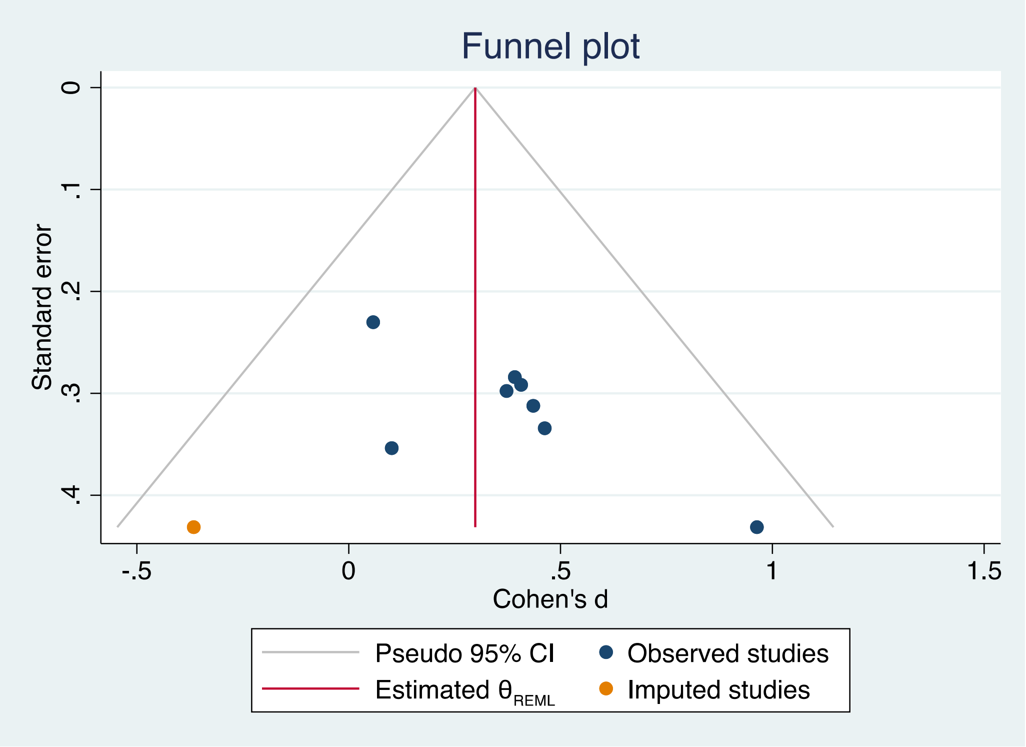

S2. Analysis for inhibition control.

Figure S3. Funnel plot of inhibition control.

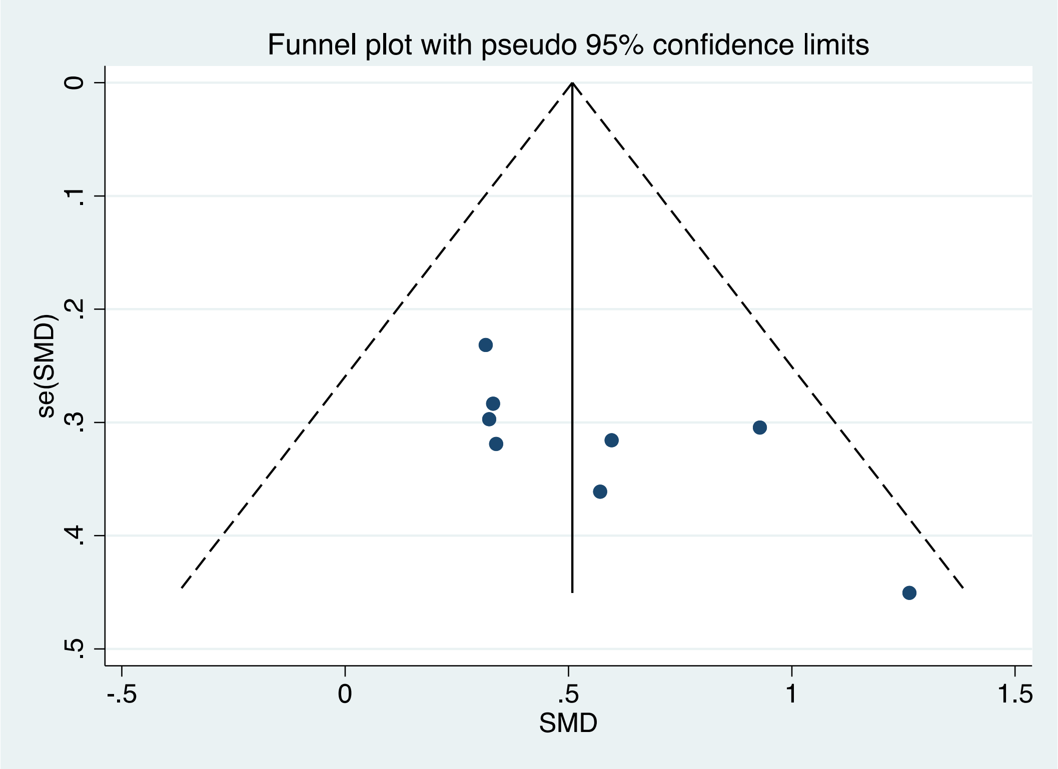

S3. Analysis for Working memory.

Figure S4. Funnel plot of Working memory.

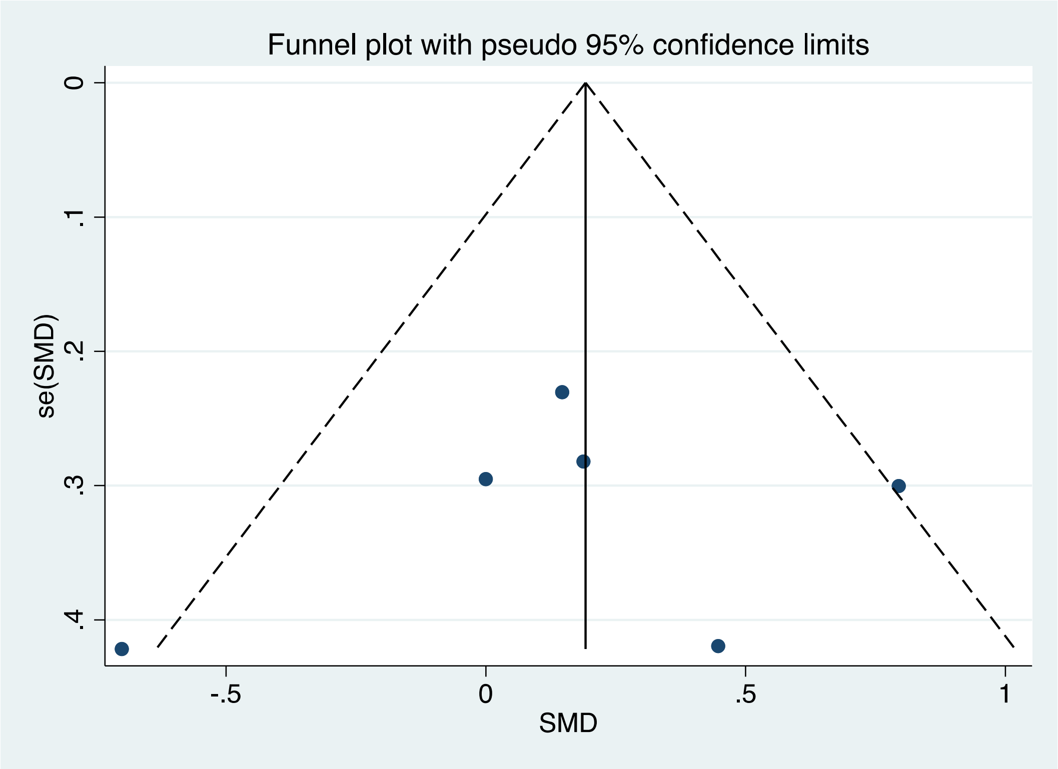

S4. Analysis for gross motor.

Figure S5. Sensitivity analysis for gross motor.

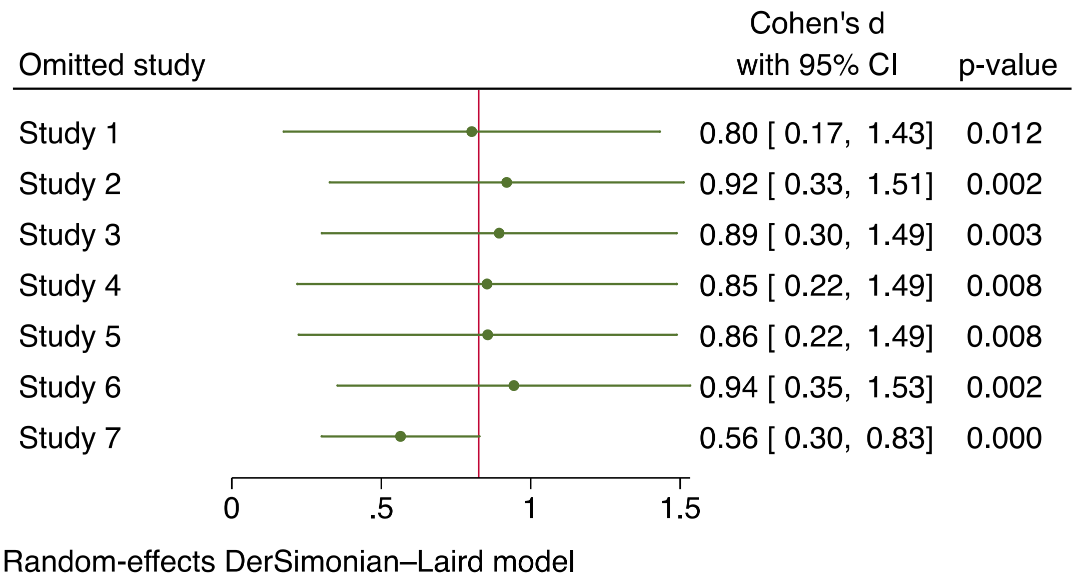

Figure S6. Forest plot after excluding 2 studies.

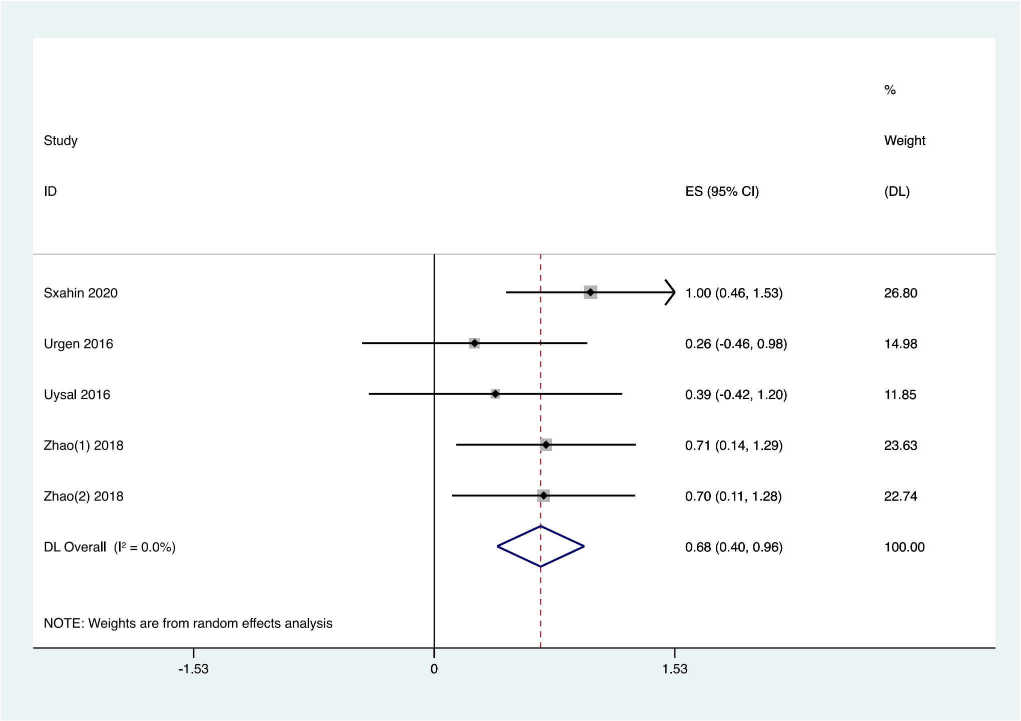

Figure S7. Funnel plot of gross motor.

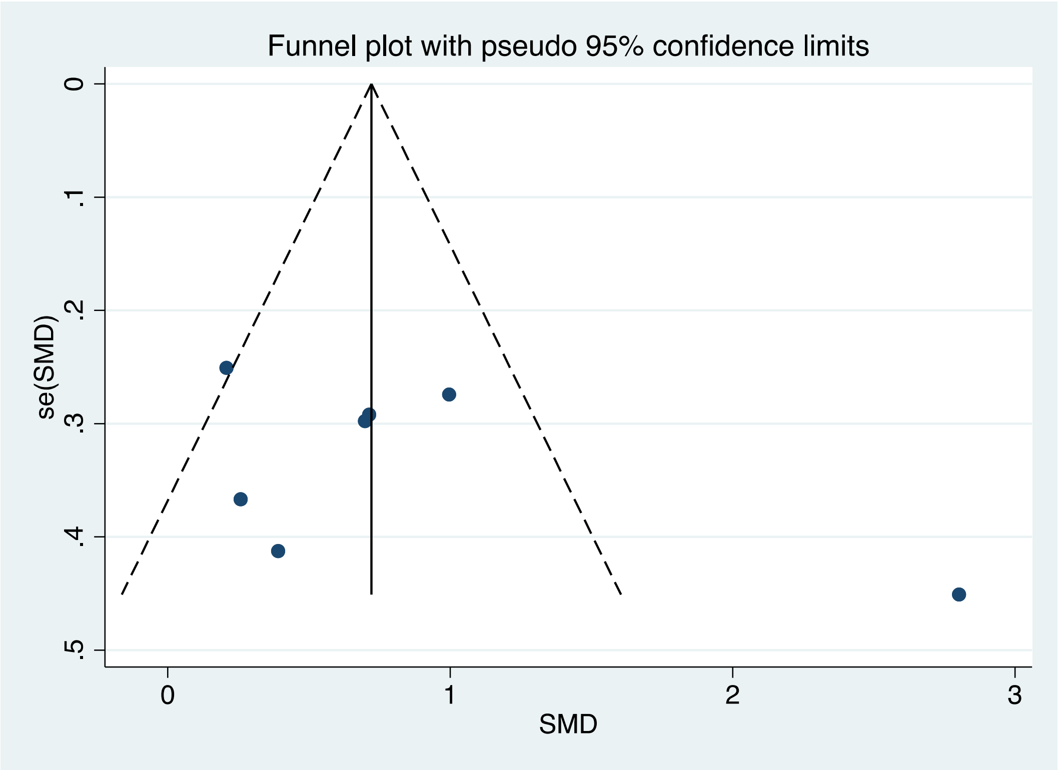

S5. Analysis for fine motor.

Figure S8. Sensitivity analysis for fine motor.

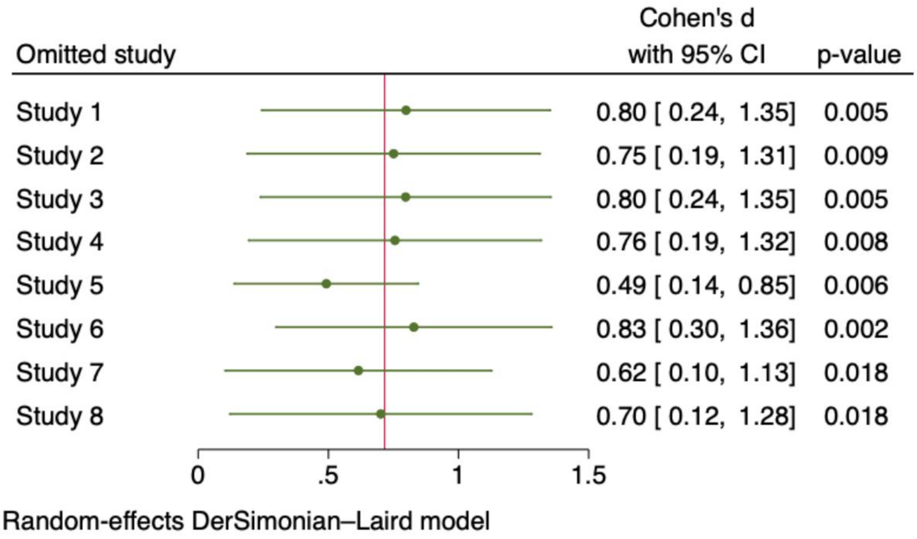

Figure S9. Forest plot after excluding 2 studies.

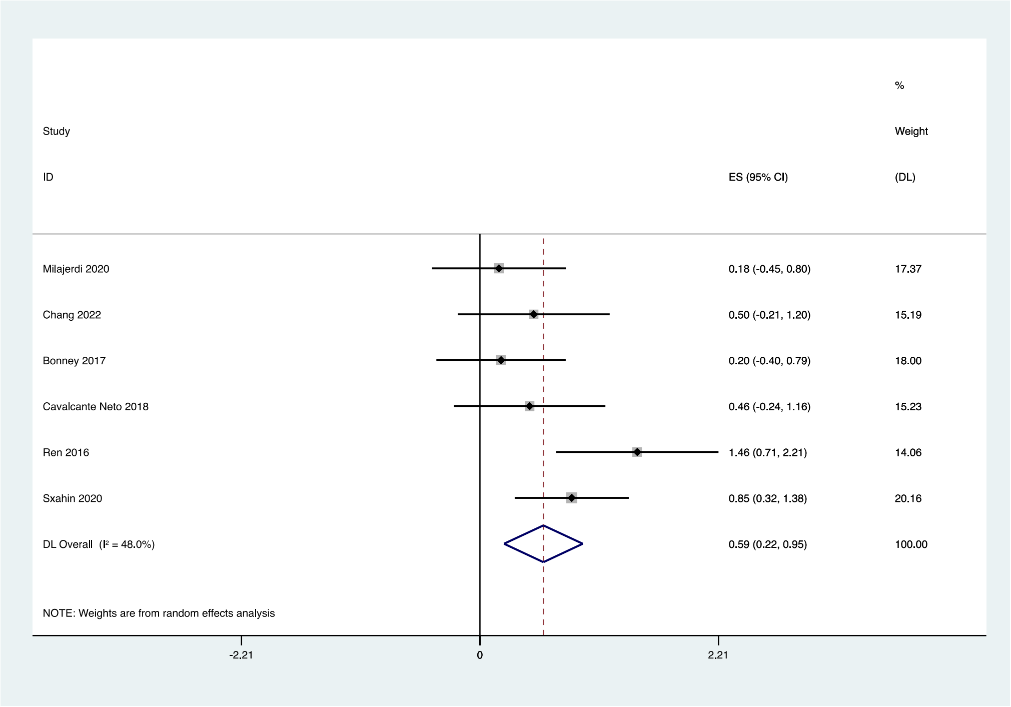

Figure S10. Funnel plot of fine motor.

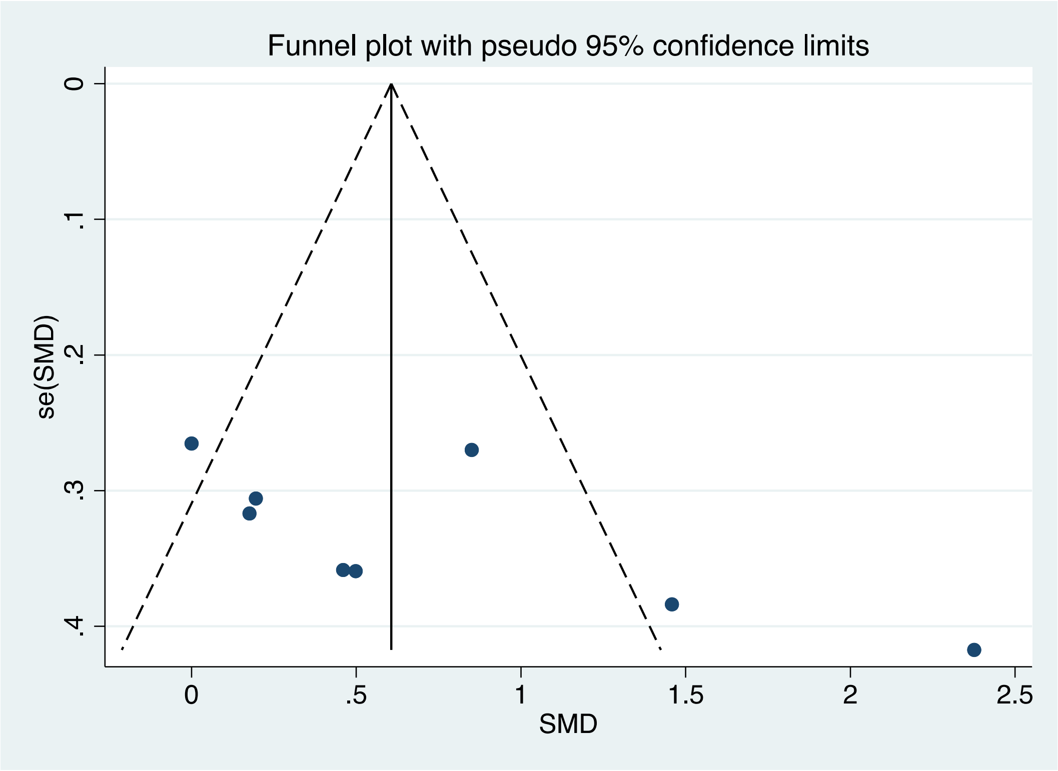

S6. Analysis for balance.

Figure S11. Sensitivity analysis for balance.

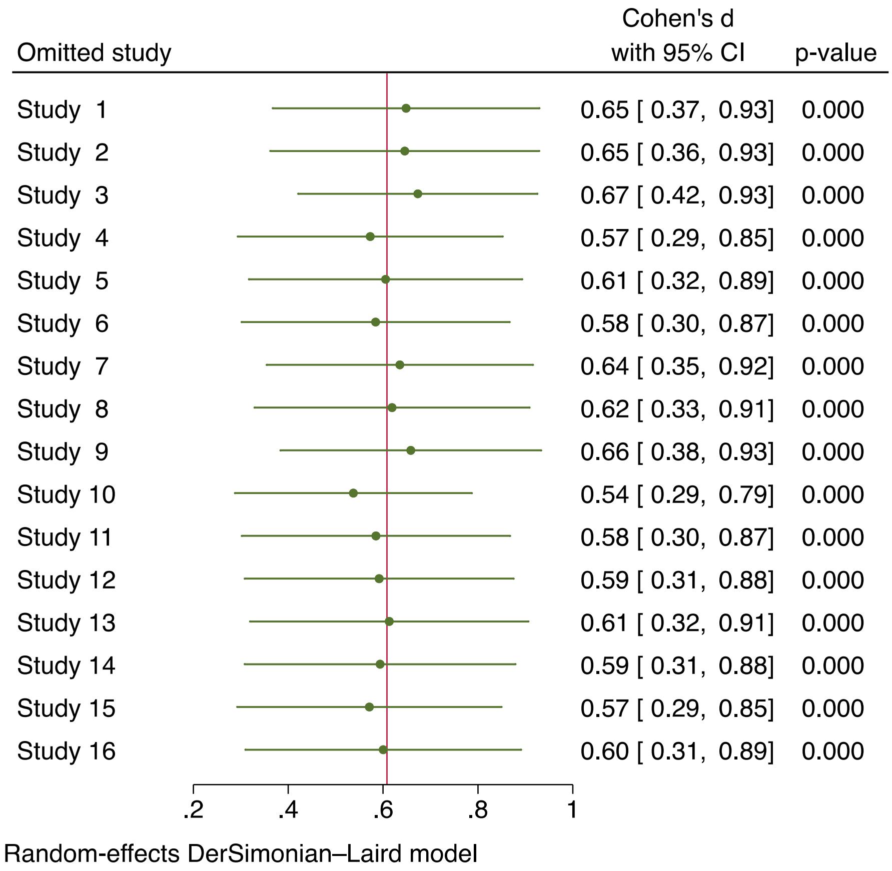

Figure S12. Forest plot after excluding 2 studies.

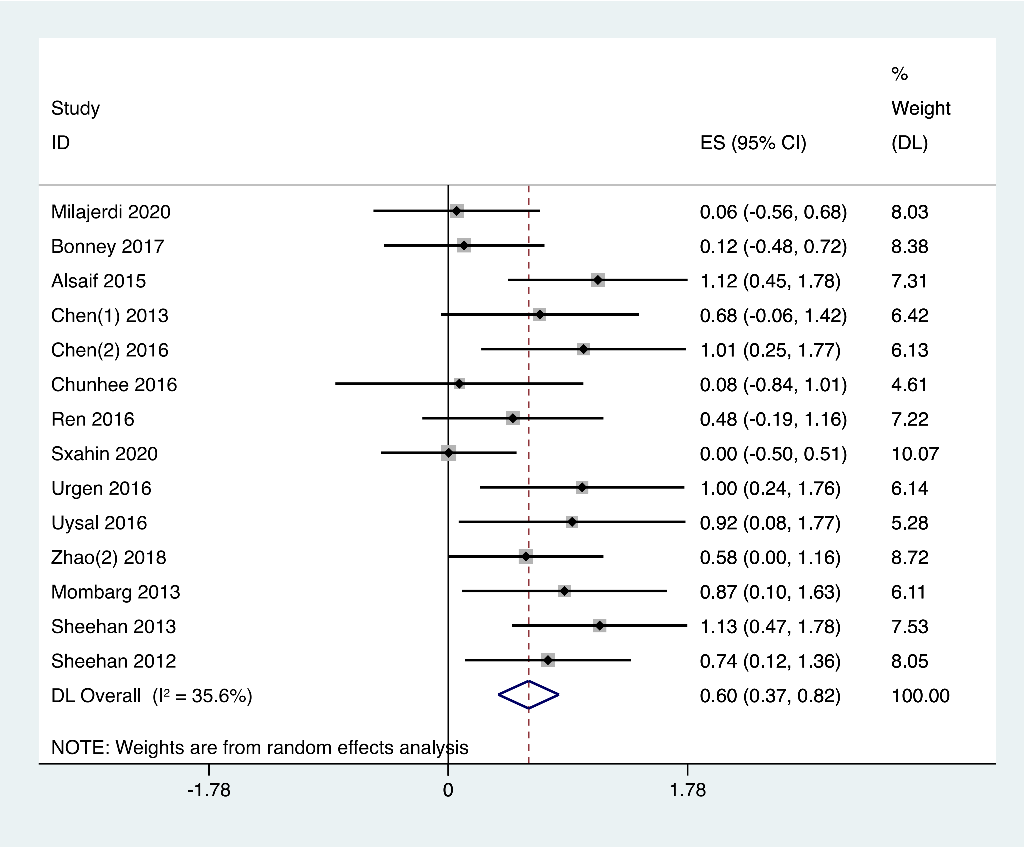

Figure S13. Funnel plot of balance.

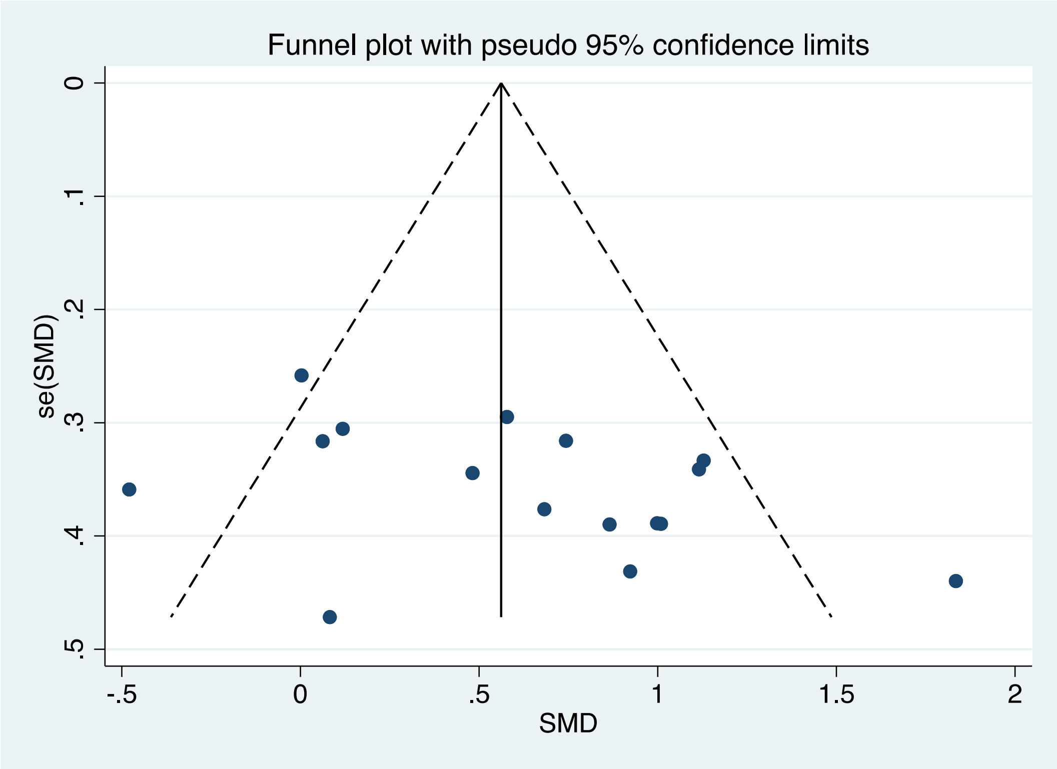

S7. Analysis for cardiorespiratory.

Figure S14. Sensitivity analysis for cardiorespiratory.

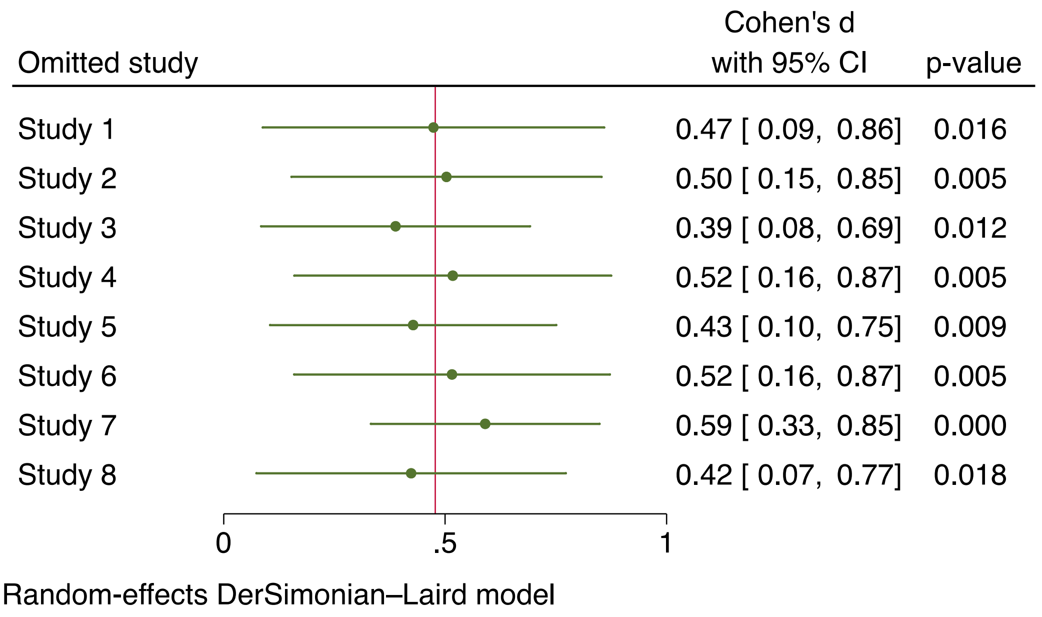

Figure S15. Forest plot after excluding 1 study (study 7).

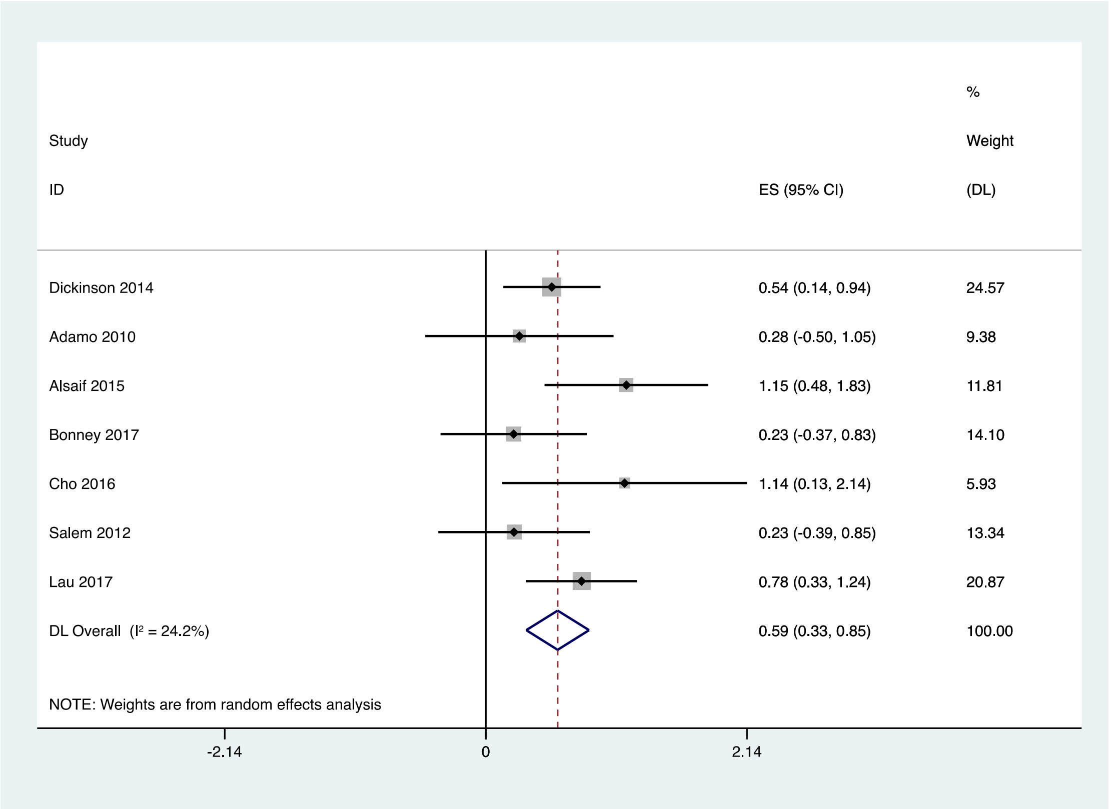

Figure S16. Funnel plot of cardiorespiratory.

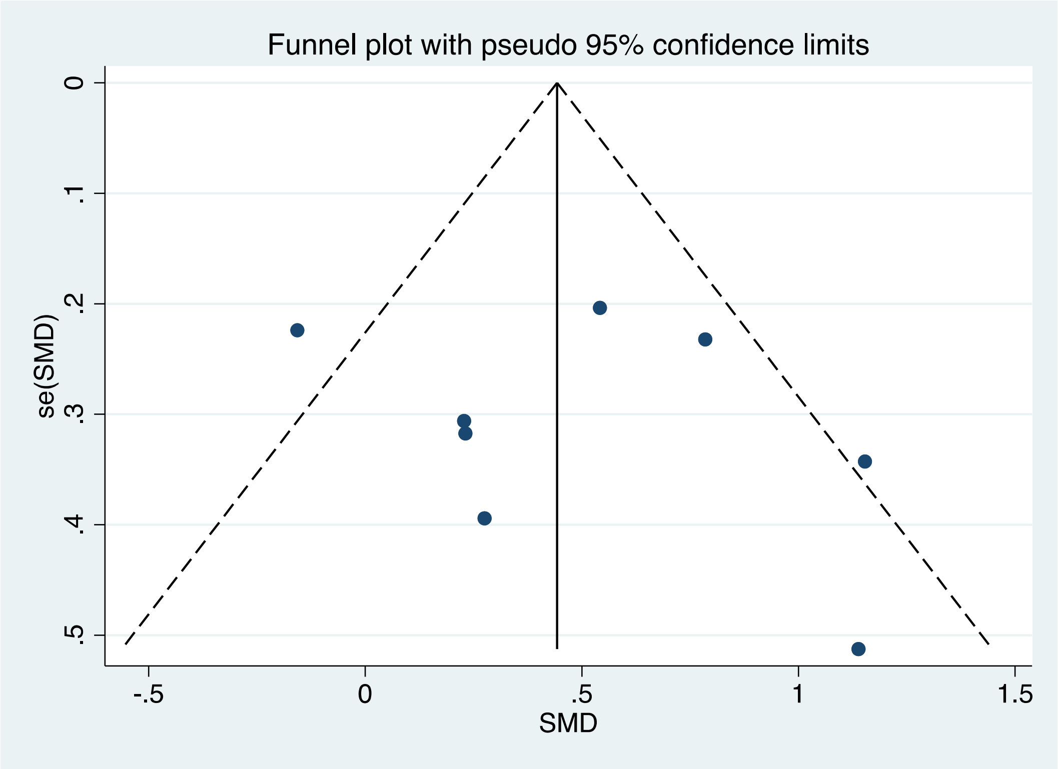

Supplement: S1 File — (PDF) [file pone.0309462.s002.pdf]
