# Supplementary material for: Effects of Exergaming on executive function and motor ability in children: A systematic review and meta-analysis
Source: PLoS One. 2024 Sep 6;19(9):e0309462. doi: 10.1371/journal.pone.0309462 (PMC11379181; doi:10.1371/journal.pone.0309462)
Supplement: S3 File — (PDF) [file pone.0309462.s004.pdf]

National Institute for Health Research

UNIVERSITY of York
Centre for Reviews and Dissemination

??For those soon to register with PROSPERO: records may not be submitted between the 22nd Dec - 2nd Jan whilst staff are away and the university is closed for the Christmas period. During this time you may still work on your record and save it for submission in the new year. ??

Systematic review

A list of fields that can be edited in an update can be found here

1. * Review title.

Give the title of the review in English

Effects of Active Video Game on motor and cognitive function in children: a systematic review and meta-analysis.

2. Original language title.

For reviews in languages other than English, give the title in the original language. This will be displayed with the English language title.

???

3. * Anticipated or actual start date.

Give the date the systematic review started or is expected to start.

11/11/2023

4. * Anticipated completion date.

Give the date by which the review is expected to be completed.

31/12/2023

5. * Stage of review at time of this submission.

This field uses answers to initial screening questions. It cannot be edited until after registration.

Tick the boxes to show which review tasks have been started and which have been completed.

Update this field each time any amendments are made to a published record.

The review has not yet started: No

Review stage	Started	Completed
Preliminary searches	Yes	No
Piloting of the study selection process	No	No
Formal screening of search results against eligibility criteria	No	No
Data extraction	No	No
Risk of bias (quality) assessment	No	No
Data analysis	No	No

Provide any other relevant information about the stage of the review here.

I've read a lot of research and collected some data.

I've read a lot of research and collected some data.

6. * Named contact.

The named contact is the guarantor for the accuracy of the information in the register record. This may be any member of the review team.

kou ruijie

Email salutation (e.g. "Dr Smith" or "Joanne") for correspondence:

Mr ruijie

7. * Named contact email.

Give the electronic email address of the named contact.

582049380@qq.com

8. Named contact address

Give the full institutional/organisational postal address for the named contact.

Capital University of Physical Education And Sports, No. 11 North Third Ring Road, Haidian District, Beijing

9. Named contact phone number.

Give the telephone number for the named contact, including international dialling code.

+8613384982053

10. * Organisational affiliation of the review.

Full title of the organisational affiliations for this review and website address if available. This field may be completed as 'None' if the review is not affiliated to any organisation.

Capital University of Physical Education And Sports

Organisation web address:

https://www.cupes.edu.cn

11. * Review team members and their organisational affiliations.

Give the personal details and the organisational affiliations of each member of the review team. Affiliation refers to groups or organisations to which review team members belong. **NOTE: email and country now MUST be entered for each person, unless you are amending a published record.**

Mr kou ruijie. Capital University of Physical Education And Sports
Ms tang yanli. Capital University of Physical Education And Sports

12. * Funding sources/sponsors.

Details of the individuals, organizations, groups, companies or other legal entities who have funded or sponsored the review.

None

Grant number(s)

State the funder, grant or award number and the date of award

None

13. * Conflicts of interest.

List actual or perceived conflicts of interest (financial or academic).

None

14. Collaborators.

Give the name and affiliation of any individuals or organisations who are working on the review but who are not listed as review team members. **NOTE: email and country must be completed for each person, unless you are amending a published record.**

15. * Review question.

State the review question(s) clearly and precisely. It may be appropriate to break very broad questions down into a series of related more specific questions. Questions may be framed or refined using PI(E)COS or

similar where relevant.

Being physically inactive or failure to meet the daily physical activity (PA) recommendations is considered to be one of the leading risk factors for many chronic diseases, physical inactivity had become the fourth leading cause of death worldwide.Active Video Game has the potential to improve motor and cognitive function in children. More studies with rigorous designs are warranted to explore the specific effects of Active Video Game intervention. The subjects of this systematic review were healthy and special needs children, the intervention was active video gaming, the control was no intervention, regular exercise, or other interventions (e.g., medication), and the outcome variables were motor and cognitive function; the study method was a randomized controlled trial.

16. * Searches.

State the sources that will be searched (e.g. Medline). Give the search dates, and any restrictions (e.g. language or publication date). Do NOT enter the full search strategy (it may be provided as a link or attachment below.)

The electronic databases of China National Knowledge Infrastructure (CNKI, in Chinese), Wan Fang (in Chinese), Web of Science (in English), Embase (in English), and PubMed (in English) were searched for studies investigating the influence of Active Video Game on children' motor and cognitive function. The publication time of articles was from inception until Nov.11, 2023. The key search terms were ("exergame" OR "active video game" OR "video game") AND "child" AND ("motor function" OR "cognitive functions" OR "inhibition control" OR "working memory" OR "cognitive flexibility").

17. URL to search strategy.

Upload a file with your search strategy, or an example of a search strategy for a specific database, (including the keywords) in pdf or word format. In doing so you are consenting to the file being made publicly accessible. Or provide a URL or link to the strategy. Do NOT provide links to your search **results**.

Alternatively, upload your search strategy to CRD in pdf format. Please note that by doing so you are consenting to the file being made publicly accessible.

Do not make this file publicly available until the review is complete

18. * Condition or domain being studied.

Give a short description of the disease, condition or healthcare domain being studied in your systematic review.

Being physically inactive or failure to meet the daily physical activity (PA) recommendations is considered to be one of the leading risk factors for many chronic diseases, physical inactivity had become the fourth leading cause of death worldwide.

19. * Participants/population.

Specify the participants or populations being studied in the review. The preferred format includes details of both inclusion and exclusion criteria.

Participants are children or adolescents aged under 18 years.

20. * Intervention(s), exposure(s).

Give full and clear descriptions or definitions of the interventions or the exposures to be reviewed. The preferred format includes details of both inclusion and exclusion criteria.

The primary intervention is active video game using any modality.

21. * Comparator(s)/control.

Where relevant, give details of the alternatives against which the intervention/exposure will be compared (e.g. another intervention or a non-exposed control group). The preferred format includes details of both inclusion and exclusion criteria.

The control groups receive no intervention, conventional exercise, or other interventions (eg, medications).

22. * Types of study to be included.

Give details of the study designs (e.g. RCT) that are eligible for inclusion in the review. The preferred format includes both inclusion and exclusion criteria. If there are no restrictions on the types of study, this should be stated.

Randomized controlled trials (RCTs) will be included.

23. Context.

Give summary details of the setting or other relevant characteristics, which help define the inclusion or exclusion criteria.

24. * Main outcome(s).

Give the pre-specified main (most important) outcomes of the review, including details of how the outcome is defined and measured and when these measurement are made, if these are part of the review inclusion criteria.

The outcome measures were motor function, cognitive function and cognitive flexibility. We have included the studies reporting any of the outcomes mentioned above in both the arms.

Measures of effect

Please specify the effect measure(s) for you main outcome(s) e.g. relative risks, odds ratios, risk difference, and/or 'number needed to treat.

MD

25. * Additional outcome(s).

List the pre-specified additional outcomes of the review, with a similar level of detail to that required for main outcomes. Where there are no additional outcomes please state ‘None’ or ‘Not applicable’ as appropriate to the review

None

Measures of effect

Please specify the effect measure(s) for you additional outcome(s) e.g. relative risks, odds ratios, risk difference, and/or 'number needed to treat.

26. * Data extraction (selection and coding).

Describe how studies will be selected for inclusion. State what data will be extracted or obtained. State how this will be done and recorded.

Two researchers scan the titles and abstracts independently, and studies that satisfied the inclusion criteria are retrieved for full-text assessment. Differences between the 2 researchers are resolved through discussion. If agreement could not be reached, a final decision was made by discussion with a third researcher.

The data are independently extracted by 2 researchers. The extracted data related to the document characteristics (first author, publication year, and country) participant characteristics (number and age of participants in the experimental and control groups), and interventions (intervention content, frequency, and duration, and measurement tools). If there are multiple control groups in a study, only the data for the control group receiving standard treatment is extracted.

27. * Risk of bias (quality) assessment.

State which characteristics of the studies will be assessed and/or any formal risk of bias/quality assessment tools that will be used.

The Cochrane Collaboration's tool for assessing risk of bias is used to categorize the risk of bias in 7 domains: sequence generation, allocation concealment, blinding of assessors, incomplete outcome data, selective outcome reporting, and other sources of bias. Items were rated as having high, low, or unclear risk of bias. Based on the risk of bias in individual domains, studies were classified as having low, unclear, or high risk of bias (green: low risk of bias; yellow: unclear risk of bias; red: high risk of bias). Disagreements on the risk of bias are resolved by discussion or by consulting a third researcher. The risk of bias for blinding of outcome assessment is based on the method of outcome assessment (objective or subjective).

28. * Strategy for data synthesis.

Describe the methods you plan to use to synthesise data. This **must not be generic text** but should be **specific to your review** and describe how the proposed approach will be applied to your data. If meta-analysis is planned, describe the models to be used, methods to explore statistical heterogeneity, and software package to be used.

The meta-analysis is performed using Review Manager (RevMan 5.3). The random effects and fixed effects models were used to calculate effect sizes. If all studies included in the analysis shared a common true

effect; we chose the fixed effects model to calculate effect sizes; otherwise, we chose the random effects model. Standardized mean differences (SMDs) with 95% confidence intervals (CIs) were calculated. The recommended by the Cochrane Handbook. SMD less than 0.20 correlated with a negligible effect, SMD between 0.20 and 0.50 correlated with a small effect, SMD between 0.50 and 0.80 correlated with a medium effect, and SMD greater than 0.80 correlated with a large effect. Heterogeneity (I^2) between the included studies was evaluated and graded (very low, 25%; low, 25%-50%; moderate, 50%-75%; high, ?75%).34 When the result is moderate or high heterogeneity, subgroup analysis is performed.34 The funnel plot of each result was processed to evaluate publication bias.

29. * Analysis of subgroups or subsets.

State any planned investigation of ‘subgroups’. Be clear and specific about which type of study or participant will be included in each group or covariate investigated. State the planned analytic approach.

In order to explore the influence of other variables on the results, a subgroup analysis was conducted based on continuous variables (including age, duration of intervention), intervention object, exercise intensity and exercise cycle.

30. * Type and method of review.

Select the type of review, review method and health area from the lists below.

Type of review

Cost effectiveness

No

Diagnostic

No

Epidemiologic

No

Individual patient data (IPD) meta-analysis

No

Intervention

No

Living systematic review

No

Meta-analysis

No

Methodology

No

Narrative synthesis

No

Network meta-analysis
No

Pre-clinical
No

Prevention
No

Prognostic
No

Prospective meta-analysis (PMA)
No

Review of reviews
No

Service delivery
No

Synthesis of qualitative studies
No

Systematic review
Yes

Other
No

Health area of the review

Alcohol/substance misuse/abuse
No

Blood and immune system
No

Cancer
No

Cardiovascular
No

Care of the elderly
No

Child health
Yes

Complementary therapies

No

COVID-19

No

Crime and justice

No

Dental

No

Digestive system

No

Ear, nose and throat

No

Education

No

Endocrine and metabolic disorders

No

Eye disorders

No

General interest

No

Genetics

No

Health inequalities/health equity

No

Infections and infestations

No

International development

No

Mental health and behavioural conditions

Yes

Musculoskeletal

Yes

Neurological

No

Nursing

No

Obstetrics and gynaecology

No

Oral health
No

Palliative care
No

Perioperative care
No

Physiotherapy
No

Pregnancy and childbirth
No

Public health (including social determinants of health)
No

Rehabilitation
No

Respiratory disorders
No

Service delivery
No

Skin disorders
No

Social care
No

Surgery
No

Tropical Medicine
No

Urological
No

Wounds, injuries and accidents
No

Violence and abuse
No

31. Language.

Select each language individually to add it to the list below, use the bin icon to remove any added in error.

English

There is an English language summary.

32. * Country.

Select the country in which the review is being carried out. For multi-national collaborations select all the countries involved.

China

33. Other registration details.

Name any other organisation where the systematic review title or protocol is registered (e.g. Campbell, or The Joanna Briggs Institute) together with any unique identification number assigned by them. If extracted data will be stored and made available through a repository such as the Systematic Review Data Repository (SRDR), details and a link should be included here. If none, leave blank.

34. Reference and/or URL for published protocol.

If the protocol for this review is published provide details (authors, title and journal details, preferably in Vancouver format)

Add web link to the published protocol.

Or, upload your published protocol here in pdf format. Note that the upload will be publicly accessible.

No I do not make this file publicly available until the review is complete

Please note that the information required in the PROSPERO registration form must be completed in full even if access to a protocol is given.

35. Dissemination plans.

Do you intend to publish the review on completion?

No

Give brief details of plans for communicating review findings.?

36. Keywords.

Give words or phrases that best describe the review. Separate keywords with a semicolon or new line. Keywords help PROSPERO users find your review (keywords do not appear in the public record but are included in searches). Be as specific and precise as possible. Avoid acronyms and abbreviations unless these are in wide use.

active video game; children; physical activity; motor and cognitive function.

37. Details of any existing review of the same topic by the same authors.

If you are registering an update of an existing review give details of the earlier versions and include a full bibliographic reference, if available.

38. * Current review status.

Update review status when the review is completed and when it is published.New registrations must be ongoing so this field is not editable for initial submission.

Please provide anticipated publication date

Review_Ongoing

39. Any additional information.

Provide any other information relevant to the registration of this review.

Incorporating more comprehensive randomized controlled trials,\nAnd there are new innovations.

40. Details of final report/publication(s) or preprints if available.

Leave empty until publication details are available OR you have a link to a preprint (NOTE: this field is not editable for initial submission). List authors, title and journal details preferably in Vancouver format.

Give the link to the published review or preprint.
