# Supplementary material for: Effects of Exergaming on executive function and motor ability in children: A systematic review and meta-analysis
Source: PLoS One. 2024 Sep 6;19(9):e0309462. doi: 10.1371/journal.pone.0309462 (PMC11379181; doi:10.1371/journal.pone.0309462)
Supplement: S1 Table — (DOCX) [file pone.0309462.s005.docx]

Detailed of Search Strategy

| Database | Search strategy |
| --- | --- |
| Web of science: 2117  Date: January 26, 2024 | ((TS=(Exergaming or exergaming or Active-Video Gaming or Active Video Gaming or Active-Video gatings or Gaming, Active-Video or gatings, Active-Video or Virtual Reality Exercise or Exercise, Virtual Reality or Exercises, Virtual Reality or Virtual Reality Exercises or exergames or exergames or Interactive Video Games or IVG or VR or Virtual Reality or Reality, Virtual or Virtual Reality, Educational or Educational Virtual Realities or Educational Virtual Reality or Reality, Educational Virtual or Virtual Realities, Educational or Virtual Reality, Instructional or Instructional Virtual Realities or Instructional Virtual Reality or Realities, Instructional Virtual or Reality, Instructional Virtual or Virtual Realities, Instructional or AR or exercuse  or Virtual reality training or Motion-Sensing Games or Kinect xbox or Wii sport  or Wii Fit or Kinect Sport   or Nintendo Switch Sports（NS Sport） or Xbox Kinect or Nintendo Wii or Computer-Based Activity )) AND TS=(Child or Children or Adolescent or Adolescents or Adolescence or Teens or Teen or Teenagers or Teenager or Youth or Youths or Adolescents, Female or Adolescent, Female or Female Adolescent or Female Adolescents or Adolescents, Male or Adolescent, Male or Male Adolescent or Male Adolescents)) AND AB=(randomized controlled trial or randomized or placebo or randomised or random or rct) |
| Cochrane Library: 1838  Date: January 26, 2024 | #1：(Exergaming):ti,ab,kw OR (Exergamings):ti,ab,kw OR (Active-Video Gaming):ti,ab,kw OR (Active Video Gaming):ti,ab,kw OR (Active-Video Gamings):ti,ab,kw OR (Gaming, Active-Video):ti,ab,kw OR (Gamings, Active-Video):ti,ab,kw OR (Virtual Reality Exercise):ti,ab,kw OR (Exercise, Virtual Reality):ti,ab,kw OR (Exercises, Virtual Reality):ti,ab,kw OR (Virtual Reality Exercises):ti,ab,kw OR (Exergames):ti,ab,kw OR (Exergame):ti,ab,kw OR (Interactive Video Games):ti,ab,kw OR (IVG):ti,ab,kw OR (VR):ti,ab,kw OR (Virtual Reality):ti,ab,kw OR (Reality, Virtual):ti,ab,kw OR (Virtual Reality, Educational):ti,ab,kw OR (Educational Virtual Realities):ti,ab,kw OR (Educational Virtual Reality):ti,ab,kw OR (Reality, Educational Virtual):ti,ab,kw OR (Virtual Realities, Educational):ti,ab,kw OR (Virtual Reality, Instructional):ti,ab,kw OR (Instructional Virtual Realities):ti,ab,kw OR (Instructional Virtual Reality):ti,ab,kw OR (Realities, Instructional Virtual):ti,ab,kw OR (Reality, Instructional Virtual):ti,ab,kw OR (Virtual Realities, Instructional):ti,ab,kw OR (AR):ti,ab,kw OR (ExerCube ):ti,ab,kw OR (Virtual reality training):ti,ab,kw OR (Motion-Sensing Games):ti,ab,kw OR (Kinect xbox):ti,ab,kw OR (Wii sport ):ti,ab,kw OR (Wii Fit):ti,ab,kw OR (Kinect Sport  ):ti,ab,kw OR (Nintendo Switch Sports（NS Sport）):ti,ab,kw OR (Xbox Kinect):ti,ab,kw OR (Nintendo Wii):ti,ab,kw OR (Computer-Based Activity ):ti,ab,kw  #2: (randomized controlled trial):ti,ab,kw OR (randomized):ti,ab,kw OR (placebo):ti,ab,kw OR (randomised):ti,ab,kw OR (random):ti,ab,kw OR (rct):ti,ab,kw  #3: (Child):ti,ab,kw OR (Children):ti,ab,kw OR (Adolescent):ti,ab,kw OR (Adolescents):ti,ab,kw OR (Adolescence):ti,ab,kw OR (Teens):ti,ab,kw OR (Teen):ti,ab,kw OR (Teenagers):ti,ab,kw OR (Teenager):ti,ab,kw OR (Youth):ti,ab,kw OR (Youths):ti,ab,kw OR (Adolescents, Female):ti,ab,kw OR (Adolescent, Female):ti,ab,kw OR (Female Adolescent):ti,ab,kw OR (Female Adolescents):ti,ab,kw OR (Adolescents, Male):ti,ab,kw OR (Adolescent, Male):ti,ab,kw OR (Male Adolescent):ti,ab,kw OR (Male Adolescents):ti,ab,kw  #4: #1 and #2 and #3 |
| Pubmed: 109  Date: January 26, 2024 | ((("Exergaming"[Mesh]) OR ((((((((((((Active-Video Gaming[Title/Abstract]) OR (Active Video Gaming[Title/Abstract])) OR (Exergamings[Title/Abstract])) OR (Active-Video Gamings[Title/Abstract])) OR (Gaming, Active-Video[Title/Abstract])) OR (Gamings, Active-Video[Title/Abstract])) OR (Virtual Reality Exercise[Title/Abstract])) OR (Exercise, Virtual Reality[Title/Abstract])) OR (Exercises, Virtual Reality[Title/Abstract])) OR (Virtual Reality Exercises[Title/Abstract])) OR (Exergames[Title/Abstract])) OR (Exergame[Title/Abstract]))) AND (((((((((((Child[Title/Abstract]) OR (Children[Title/Abstract])) OR (Adolescent[Title/Abstract])) OR (Adolescents[Title/Abstract])) OR (Adolescence[Title/Abstract])) OR (Teens[Title/Abstract])) OR (Teen[Title/Abstract])) OR (Teenagers[Title/Abstract])) OR (Teenager[Title/Abstract])) OR (Youth[Title/Abstract])) OR (Youths[Title/Abstract]))) AND ((((((randomized controlled trial[Title/Abstract]) OR (randomized[Title/Abstract])) OR (placebo[Title/Abstract])) OR (randomised[Title/Abstract])) OR (random[Title/Abstract])) OR (rct[Title/Abstract])). |
| Scopus: 243  Date: January 27, 2024 | ( TITLE-ABS-KEY ( "child" OR "children" OR "adolescent" OR "adolescents" OR "adolescence" OR "teens" OR "teen" OR "teenagers" OR "teenager" OR "youth" OR "youths" OR "adolescents, female" OR "adolescent, female" OR "female adolescent" OR "female adolescents" OR "adolescents, male" OR "adolescent, male" OR "male adolescent" OR "male adolescents" ) ) AND ( TITLE-ABS-KEY ( "randomized controlled trial" OR "randomized" OR "placebo" OR "randomised" OR "random" OR "rct" ) ) AND ( TITLE-ABS-KEY ( "exergaming" OR "exergamings" OR "active-video gaming" OR "active video gaming" OR "active-video gamings" OR "gaming, active-video" OR "gamings, active-video" OR "virtual reality exercise" OR "exercise, virtual reality" OR "exercises, virtual reality" OR "virtual reality exercises" OR "exergames" OR "exergame" OR "interactive video games" OR "ivg" OR "motion-sensing games" OR "kinect xbox" OR "wii sport " OR "wii fit" OR "kinect sport " OR "nintendo switch sports（ns sport）" OR "xbox kinect" OR "nintendo wii" OR "computer-based activity " ) ) |
| Embase: 969  Date: January 27, 2024 | #1: 'exergaming':ab,ti OR 'exergamings':ab,ti OR 'active-video gaming':ab,ti OR 'active video gaming':ab,ti OR 'active-video gamings':ab,ti OR 'gaming, active-video':ab,ti OR 'gamings, active-video':ab,ti OR 'virtual reality exercise':ab,ti OR 'exercise, virtual reality':ab,ti OR 'exercises, virtual reality':ab,ti OR 'virtual reality exercises':ab,ti OR 'exergames':ab,ti OR 'exergame':ab,ti OR 'interactive video games':ab,ti OR 'ivg':ab,ti OR 'vr':ab,ti OR 'virtual reality':ab,ti OR 'reality, virtual':ab,ti OR 'virtual reality, educational':ab,ti OR 'educational virtual realities':ab,ti OR 'educational virtual reality':ab,ti OR 'reality, educational virtual':ab,ti OR 'virtual realities, educational':ab,ti OR 'virtual reality, instructional':ab,ti OR 'instructional virtual realities':ab,ti OR 'instructional virtual reality':ab,ti OR 'realities, instructional virtual':ab,ti OR 'reality, instructional virtual':ab,ti OR 'virtual realities, instructional':ab,ti OR 'ar':ab,ti OR 'exercube ':ab,ti OR 'virtual reality training':ab,ti OR 'motion-sensing games':ab,ti OR 'kinect xbox':ab,ti OR 'wii sport ':ab,ti OR 'wii fit':ab,ti OR 'kinect sport ':ab,ti OR 'nintendo switch sports（ns sport）':ab,ti OR 'xbox kinect':ab,ti OR 'nintendo wii':ab,ti OR 'computer-based activity ':ab,ti  #2: 'child':ab,ti OR 'children':ab,ti OR 'adolescent':ab,ti OR 'adolescents':ab,ti OR 'adolescence':ab,ti OR 'teens':ab,ti OR 'teen':ab,ti OR 'teenagers':ab,ti OR 'teenager':ab,ti OR 'youth':ab,ti OR 'youths':ab,ti  #3: 'randomized controlled trial':ab,ti OR 'randomized':ab,ti OR 'placebo':ab,ti OR 'randomised':ab,ti OR 'random':ab,ti OR 'rct':ab,ti  #4: #1 AND #2 AND #3 |
