# Supplementary material for: Effects of Exergaming on executive function and motor ability in children: A systematic review and meta-analysis
Source: PLoS One. 2024 Sep 6;19(9):e0309462. doi: 10.1371/journal.pone.0309462 (PMC11379181; doi:10.1371/journal.pone.0309462)
Supplement: S2 Table — (DOCX) [file pone.0309462.s006.docx]

**S2 Table. GRADE Assessment****.**

**Effects of exergaming on relevant indicators**

| Outcomes | Study  Design | Risk of Bias | Inconsistency | Indirectness | Imprecision | Other  Considerations | No. of Participants | | Absolute Effect (95% CI) | Quality |
| --- | --- | --- | --- | --- | --- | --- | --- | --- | --- | --- |
|  | | | | | | | Exergaming | Control |  |  |
| Cognitive flexibility | RCTs | Serious | Serious | No | No | Serious | 177 | 179 | SMD 0.34 (0.13 to 0.55) | Very low |
| Inhibition control | RCTs | No | No | No | Serious | No | 179 | 180 | SMD 0.51 (0.30 to 0.72) | Moderate |
| Working memory | RCTs | No | Serious | No | Serious | Serious | 133 | 136 | SMD 0.19 (-0.05 to 0.43) | Very low |
| global cognitive | RCTs | No | No | No | Serious | Serious | 66 | 64 | SMD 0.87 (0.50 to 1.23) | Low |
| Gross Motor Skills | RCTs | Serious | Serious | No | No | Serious | 158 | 158 | SMD 0.82 (0.30 to 1.35) | Very Low |
| Fine Motor Skills | RCTs | Serious | Serious | No | No | Serious | 172 | 167 | SMD 0.74 (0.32 to 1.15) | Very low |
| Balance Function | RCTs | Serious | Serious | No | No | Serious | 289 | 285 | SMD 0.56 (0.39 to 0.73) | Very low |
| Cardiorespiratory Function | RCTs | No | Serious | No | No | No | 209 | 219 | SMD 0.56 (0.37 to 0.76) | Moderate |

RCTs, randomized controlled trials. MD, mean difference. SMD, standardized mean difference.
